# Water﻿ vapor induced self-assembly of islands/honeycomb structure by secondary phase separation in polystyrene solution with bimodal molecular weight distribution

**DOI:** 10.1038/s41598-021-92594-1

**Published:** 2021-06-24

**Authors:** Maciej Łojkowski, Adrian Chlanda, Emilia Choińska, Wojciech Swieszkowski

**Affiliations:** 1 Faculty of Material Sciences and Engineering, Warsaw University of Technology, Wołoska 141, 02-507 Warsaw, Poland; 2 Department of Chemical Synthesis and Flake Graphene, Łukasiewicz Research Network - Institute of Microelectronics and Photonics, Aleja Lotników 32/46, 02-668 Warsaw, Poland; 3 Centre for Advanced Materials and Technology CEZAMAT, Warsaw University of Technology, Warsaw, Poland

**Keywords:** Surfaces, interfaces and thin films, Phase transitions and critical phenomena, Self-assembly, Fluid dynamics, Mechanical properties, Polymers, Thermodynamics, Chemical physics

## Abstract

The formation of complex structures in thin films is of interest in many fields. Segregation of polymer chains of different molecular weights is a well-known process. However, here, polystyrene with bimodal molecular weight distribution, but no additional chemical modification was used. It was proven that at certain conditions, the phase separation occurred between two fractions of bimodal polystyrene/methyl ethyl ketone solution. The films were prepared by spin-coating, and the segregation between polystyrene phases was investigated by force spectroscopy. Next, water vapour induced secondary phase separation was investigated. The introduction of moist airflow induced the self-assembly of the lower molecular weight into islands and the heavier fraction into a honeycomb. As a result, an easy, fast, and effective method of obtaining island/honeycomb morphologies was demonstrated. The possible mechanisms of the formation of such structures were discussed.

## Introduction

Complex morphologies compromising micro-islands and especially micropillars have gained attention due to their wide range of possible applications, such as their special wetting properties^1,2^, application in studying biofilm formation^3^, or controlling stem cell differentiation^4^. Two widespread methods that allow the creation of a broad range of structures of polymer thin films (PTFs) are spin-coating^5^ and breath figures^6^. These methods were applied for manufacturing organic ferroelectric switches ^7^, light-emitting devices^8^, sensors^9,10^, drug delivery systems^11,12^, biologically active surfaces^13,14^, functional nanostructured surfaces^15,16^, and membranes^17^. These processes rely heavily on the interaction dynamics between the solvent, the polymer, and the vapours in the vicinity of the surface. During spin-coating, a droplet of a mixture of a solvent and one or two polymers is dropped onto the substrate. Subsequently, the substrate is rotated very quickly to cover it uniformly with the solution's liquid film. As a result, the solvent evaporates. Thus, solvent and temperature quench occurs. The spin coating can be divided into time regions. First, the liquid solution droplet is spread hydrodynamically over the substrate. This regime is often referred to as hydrodynamic thinning. Later, when a flat layer of the solution was obtained, the evaporation of the solvent was responsible for further thinning of this layer^18^. Changes in the solvent volume fraction and temperature often lead to unintentional or intentional liquid–liquid phase separation^19^. It has been argued that such phase separation often starts in the early stages of the regime controlled by evaporation^19,20^. Further spinning of the solution leads to gel formation, which eventually slows down the diffusion inside the film. As a result, the morphology becomes frozen in time before reaching equilibrium. The time necessary for the morphology to stop evolving depended on the solvent evaporation rate, solution viscosity, spinning rate, and substrate thermal properties^21–23^. During coating formation, a range of events attributed to the local thermal instabilities takes place: heat transfer between the bottom and top layers of the film; heat transfer from the air above the layer; heating the substrate by the surrounding air; and local lateral temperature variance due to the thermal conductivity and heat capacity of the substrate^23^. These events may lead to the unnecessary or intended waviness of the coating profile. This undulation is often attributed to two interconnected phenomena. One is the flow of the liquid due to thermal convection. The second is the surface tension gradient due to local composition differences, which manifests in solvent-rich or solvent-depleted areas^18^. The solvent-depleted areas have higher surface tension and thus pull the liquid towards these areas and up, while the solvent-rich areas sink, forming valleys. High centrifugal force during spin-coating is often a cause of elongation of the mentioned structures. As a result, long stripes extending from the centre of the sample are observed. Using solvents with low surface tension, the addition of a surfactant or using a mixture of solvents can suppress the formation of these features. Furthermore, decreasing the amount of heat can suppress the formation of convection cells and allow smooth coating preparation.

In the second technique mentioned above, breath figures appear on the liquid film's surface when the humid airflow accelerates the evaporation rate. Successively, the temperature decreases, which results in nucleation and growth of water droplets. These droplets create a regular honeycomb array of cavities in the film. The temperature increased to that of the surroundings, and the droplets evaporated, leaving a porous surface. After most of the solvent had evaporated, the start of droplet nucleation was governed by the onset time related to the solvent evaporation rate, solution concentration, and airflow^24^.

The location and width of the MWD can affect solid thin polymeric film formation to achieve unique properties^25^. Wu et al. studied the effect of MWD on the self-assembly of end-functionalized polystyrenes. They proposed a new way of controlling the morphology of PTF obtained via breath figures by changing the MWD width. As a result, a porous membrane with higher robustness was obtained^26^. The width of the MWD can be tailored either within the polymerization process^27,28^ or by mixing two polymer species with a very narrow MWD^29^. Heitmiller et al. reported that a heterogeneous melt of polyethylene had a higher flow index than a homogeneous melt^30^. The investigation performed by Koningsveld et al. has shown that the bimodal MWD has a significant effect on the liquid–liquid binodal curve of polymers in solution^31^. Phase regions characterise such solutions, and liquid–liquid phase separation between polymer- and solvent-rich fractions can occur. Zeman et al. demonstrated that the critical concentration enabling phase separation in a solution of two polymer species decreases with an increase in the molecular weight M_w_^32^. Moreover, even when the polymer–polymer interactions are athermal, i.e. Flory–Huggins interaction parameter χ equals zero, and phase separation can occur due to the large difference in entropy between long and short chains, which act as separate entities and influence the viscosity of bimodal solutions^33^. The evaporation of the solvent followed by temperature quenching can be responsible for phase separation, as the Flory–Huggins parameter is temperature dependent and decreases with temperature. The quenched mixture can turn from the one-phase state to an unstable state. Interestingly, Henderson et al. investigated a two-step process in which the first temperature quench forces phase separation and the spinodal structures are allowed to coarsen; next, even deeper quenching is enacted^34^. This two-step quench led to the formation of small well-dispersed domains within the primary domains. Intriguingly, the combined spin-coating and breath figure methods would lead to two quenching and heating events. First, solvent evaporation would decrease the temperature. Second, water condensation would increase the temperature, and finally, evaporating water would again decrease the temperature. Another interesting theory is “viscoelastic phase separation”. Phase separation occurs due to an imbalance of the viscoelastic properties of the components^35^. Polymer melts with bimodal molecular weights show nonlinear rheology. A rheological investigation by Hengeller et al. reported two regimes of stress relaxation in such a melt, where either the only short or only long chains underwent stress relaxation, depending on the time scale^36^. Harris et al. found that the viscosity of the blend of bimodal polystyrene can be considered a sum of components^37^. It has been discussed that blending polystyrenes with different molecular weights mixed the entanglement types between polymer chains. The polymer concentration in the solvent changes how the polymer chains interact. Two polymer chains act as separate entities, provided that the concentration is below the overlap concentration (C*). However, once the overlap concentration occurs, the polymer's cooperative motion starts, and the behaviour of the solution changes^38,39^.

Successive research has focused on studying how polymer chains of varying lengths segregate in PTFs. Hariharan et al. investigated the effect of the entropy of spin-coated and annealed bimodal PTFs on polymer chain segregation^40^. It was shown that higher entropy of shorter chains led to their segregation on the PTF surface, while the longer chains' lower entropy promoted their segregation in bulk. Tanaka et al., in turn, studied spin-coated polystyrene blends with low and high M_w_ with narrow MWD utilizing toluene as a solvent. They reported that PTFs consisting of polystyrenes with a low molecular weight demonstrated surface segregation after thermal treatment^29^. Several other recent studies have illustrated the segregation of lower molecular mass elements towards the surface during annealing^41–45^.

On the other hand, it has been shown that deuterated polymer segregation can change the surface roughness after annealing the coating^46–49^.

In the present study, to modify the coating morphology, we decided to explore the phase separation between two kinds of polystyrene with low and high molecular weights mixed in a solvent. We changed the polydispersity, and we used uniform polystyrene standards as a reference. The solvent methyl ethyl ketone MEK was chosen because of its suitability for spin-coating^50^. However, for most, its hygroscopic properties, as we planned to perform spin-coating in humid conditions. MEK is considered a marginal solvent for PS, while it is more hygroscopic than typically used solvents for polystyrene^51,52^.

For our study, we established the following assumptions. The mixture with sufficiently high polydispersity will phase separate during spin-coating, even if these species are chemically identical. The study will be performed in the regime where thermocapillary effects cause undulations to make any morphological changes clearer to observe. The breath figure technique will be combined with spin-coating to induce additional motion caused by the surface tension of water.

The study is organized in the following order. First, the effect of the bimodal weight distribution on the viscosity of the solutions is discussed. Next, the morphology of the coatings spun at the most negligible possible humidity is compared by analysis of atomic force microscopy and optical microscopy images. Then, force spectroscopy was used to identify the different polystyrene phases. In situ reflectometry was used to measure the evaporation time of the solutions. Finally, the coatings spun at different humidity levels were investigated by optical and atomic force microscopy.

It was found that at a relative humidity of 75%, a solution concentration of 80 mg/ml and polystyrene molecular weights of 20 kDa and 200 kDa mixed in a 75/25 w/w% ratio, the two phases formed islands (lower M_w_ fraction) embedded inside of the honeycomb (higher M_w_ fraction).

Although spin-coating was chosen for its ease of controlling the evaporation rate, we believe that the proposed method can be extended to other techniques, such as dip-coating or ink-jet printing. The presented results can also be treated as a starting point for other interesting experiments.

## Result and discussion

**Figure 1:**
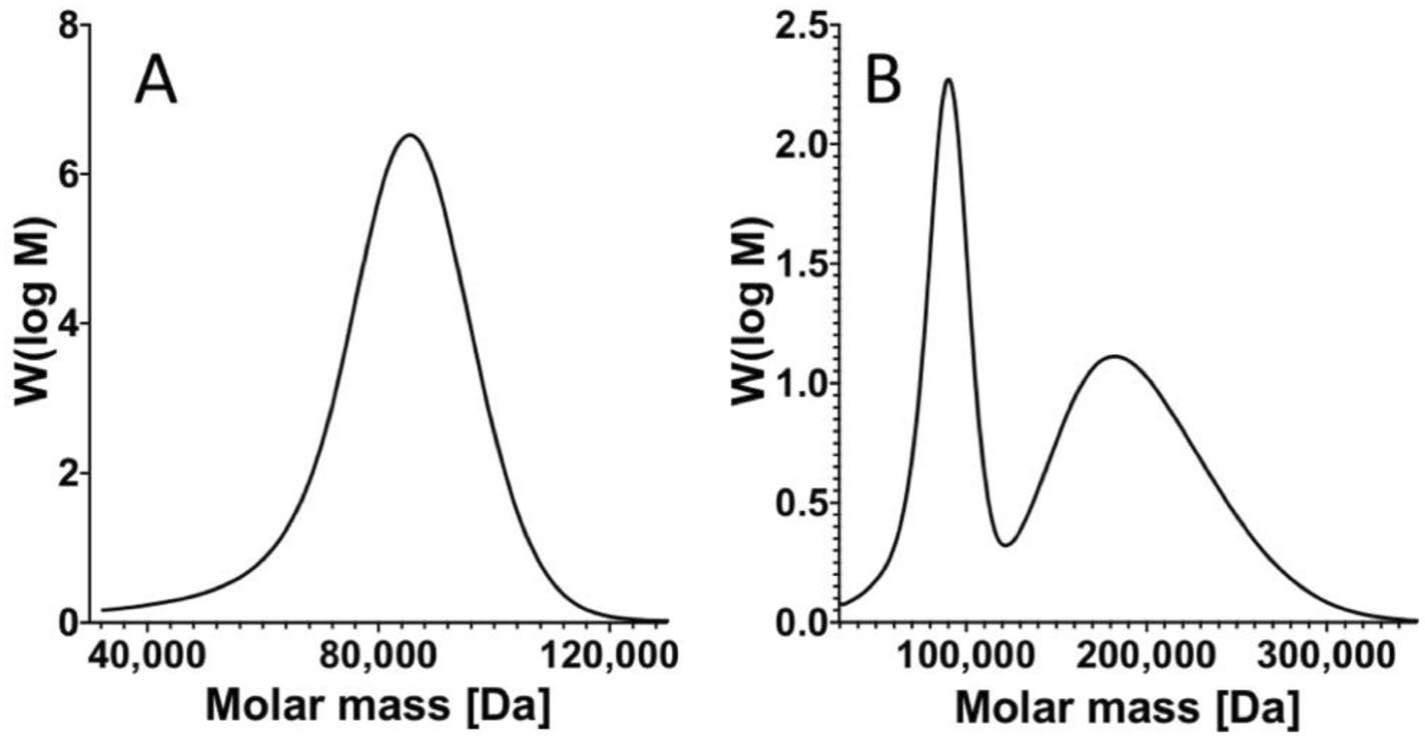
Exemplary GPC experiment results of bimodal and uniform MWD polystyrene; (**A**) narrow uniform MWD, Mw = 91 kDa, PDI 1.04; (**B**) bimodal MWD, blend of Mw = 91 kDa, PDI = 1.04 and Mw = 200 kDa, PDI = 1.04.

The solutions with bimodal MWD were prepared so that the two nodes in the distribution were clearly separated. The GPC molecular weight distribution of a single node MWD (uniform) is presented in Fig. 1A, which illustrates the MWD of 91 kDa polystyrene with a narrow distribution (PDI = 1.04). In contrast, Fig. 1B illustrates the MWD with two nodes of a blend of 91 kDa PS with 200 kDa PS, both with narrow distributions (PDI = 1.04).

### Evaluation of solution viscosities

**Figure 2:**
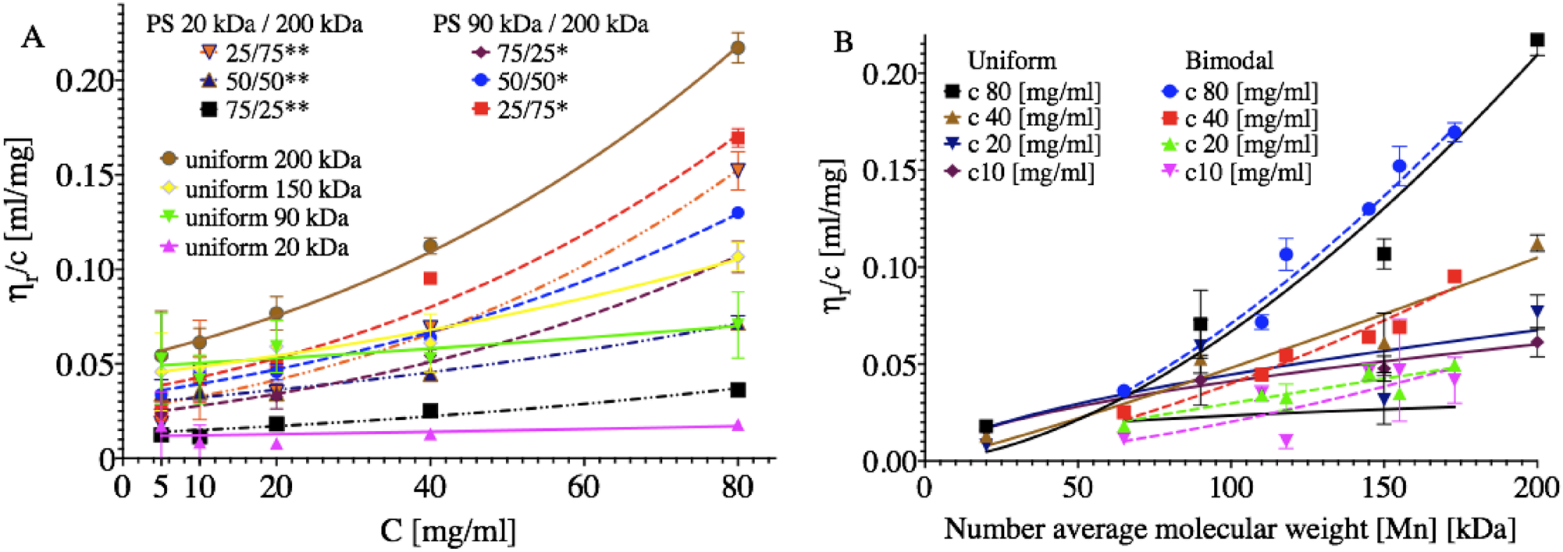
Reduced viscosity η_r_/C of the solutions, (**A**) η_r_/C plotted against the solution's concentration. Curves represent fits for Eq. 1; (**B**) η_r_/C plotted as a function of the number average molecular weight function. Curves represent the Mark-Houwink equation fit (Eq. 2), [M_n_] = *f*_*1*_*M*_*w1*_ + *f*_*2*_*M*_*w2*_, where *f* is w/w % ratio of polymers.

The viscosity measurement (Fig. 2) can be used to assess not only the final coating thickness and the solvent evaporation time prediction but also the characteristics of polymer chain interactions. The measurement result is presented as reduced viscosity η_r_/C, where C represents the concentration in mg/ml. Here, ${\rm{\eta }}_{r}=\frac{\rm{\eta }-{\rm{\eta }}_{s}}{{\rm{\eta }}_{s}}$, where η is the dynamic viscosity of the solution and η_s_ is the viscosity of the solvent. In Fig. 2A, the viscosity is plotted as a function of the concentration.

The general dependence of viscosity on concentration can be described in the form of a power series^53^:

1 $$\frac{{\eta _{r} }}{C} = \left[ \eta \right]\left( {1 + K\left[ \eta \right]C + \frac{{K\left[ \eta \right]C^{2} }}{2} + \frac{{K\left[ \eta \right]C^{3} }}{6}} \right)$$

where [η] is the intrinsic viscosity at infinite dilution. The coefficients are summarised in Table S1 in SI. The intrinsic viscosity [η] was lower for bimodal blends. The overlap concertation is defined as the concentration at which the polymer chains start to overlap with each other^54^. The overlap concertation was estimated according to C* = 1/[η]. The overlap concentration for 20 kDa was 83 mg/ml and for 200 kDa was 19 mg/ml. Notably, in the case of the 80 mg/ml solution, the 20 kDa species were in the semidilute regime, while the 200 kDa species were in the concentrated regime.

The viscosity of the bimodal solutions increased faster with increasing concentration than in the case of uniform solutions. The K parameter was particularly high for 75/25 blends: 1.36 for 90 kDa and 200 kDa and 1.01 for 20 kDa and 200 kDa. In comparison, K for uniform 200 kDa was 0.36. Thus, it can be assumed that the number of entanglements attributed to the 200 kDa fraction rises at higher concentrations. Figure 2B presents the viscosity in relation to the number molecular weight [M_n_]. The relation between viscosity and molecular weight can be described in the form of the Mark-Houwink equation^53^:

2 $$\frac{{\eta _{r} }}{C} = {\text{ln}}K + a{\text{ln}}\left[ {M_{n} } \right]$$

The *K* and [η] values are summarised in SI, Table S2. The uniform solutions were visibly more viscous than their bimodal counterparts of similar molecular weights at concentrations below 20 mg/ml. It could be expected that the bimodal solutions will change their behaviour more severely when solvent evaporation would quench them towards more concentrated regimes.

### Evaporation of the solvent during spin-coating

**Figure 3:**
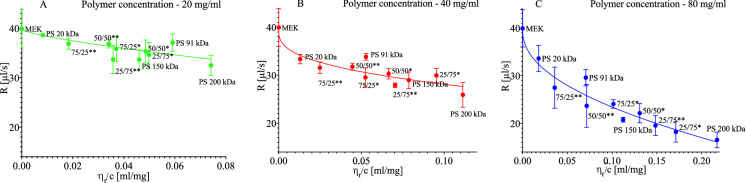
The relation between the evaporation rate of solution and the reduced viscosity of solution for polymer concentrations: (**A**) 20 mg/ml, (**B**) 40 mg/ml, and (**C**) 80 mg/ml. The curves represent the Padé approximation trend line.

As presented in Fig. 3, the evaporation rate R depends on the viscosity of the solutions. The evaporation of the 20 kDa, 91 kDa and 200 kDa samples differs for the same concentration. These differences became more transparent at a concentration of 80 mg/ml.

### Investigation of the thickness and morphology of the coatings spun at a relative humidity of 0%

The coatings spun at Rh 0% were chosen as a starting point for the investigation. We decided to look for phase separation if no humidity was applied, affecting the investigated solvent-polymer system. The convection Marangoni flow, solvent evaporation, and phase separation events alter the coating surface morphology. As a result, the occurrence of wrinkles or arrays of islands on the coating surface was reported^48,55–57^.

**Figure 4:**
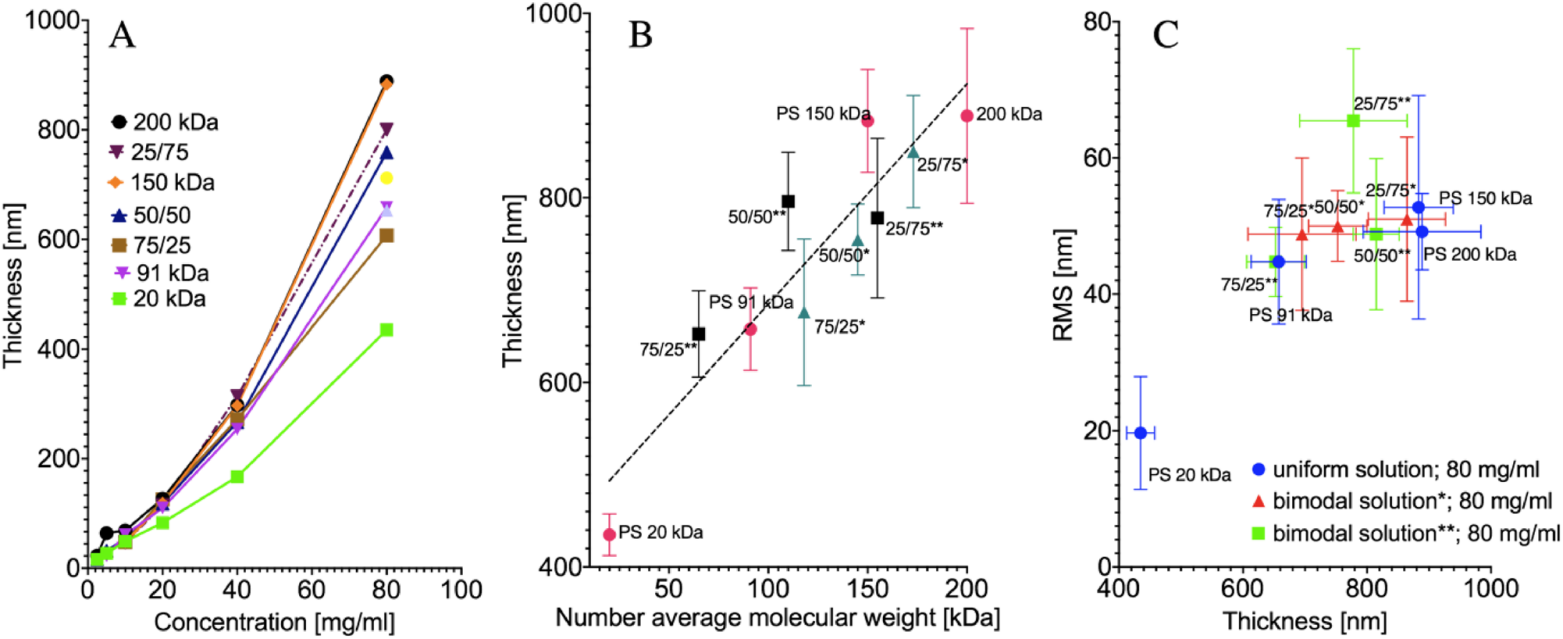
(**A**) Thickness of the coatings with respect to the composition and the concentration. The plot presents data for uniform and 91 kDa/200 kDa solutions. (**B**) Thickness of the coating for 80 mg/ml concentration in the blend’s molecular weight function. *Blends of 91 kDa and 200 kDa polystyrene; **blend of 20 kDa and 200 kDa polystyrene; x/x – w/w% ratio of blended homogeneous polystyrenes. The number average molecular weight [M_n_] = *f*_*1*_*M*_*w1*_ + *f*_*2*_*M*_*w2*_, where *f* w/w. % ratio of polymers. (**C**) RMS roughness of the coatings spun from 80 mg/ml concentration.

The coatings were investigated via AFM and optical imaging to determine the effect of bimodal MWD on coating morphology. Significant differences between blend types occurred (Fig. 4A). The solution concentration of 80 mg/ml was chosen. As we expected from the tests mentioned earlier, the bimodal distribution role would be the highest. Moreover, we wanted to avoid the influence of the substrate on our force spectroscopy experiment. For that, we needed the thickest coating. As illustrated in Fig. 4B, the thickness of the coatings in the case of 80 mg/ml scaled linearly with the blends' average molecular weight. No correlation between the thickness and roughness was found. (Fig. 4C).

**Figure 5:**
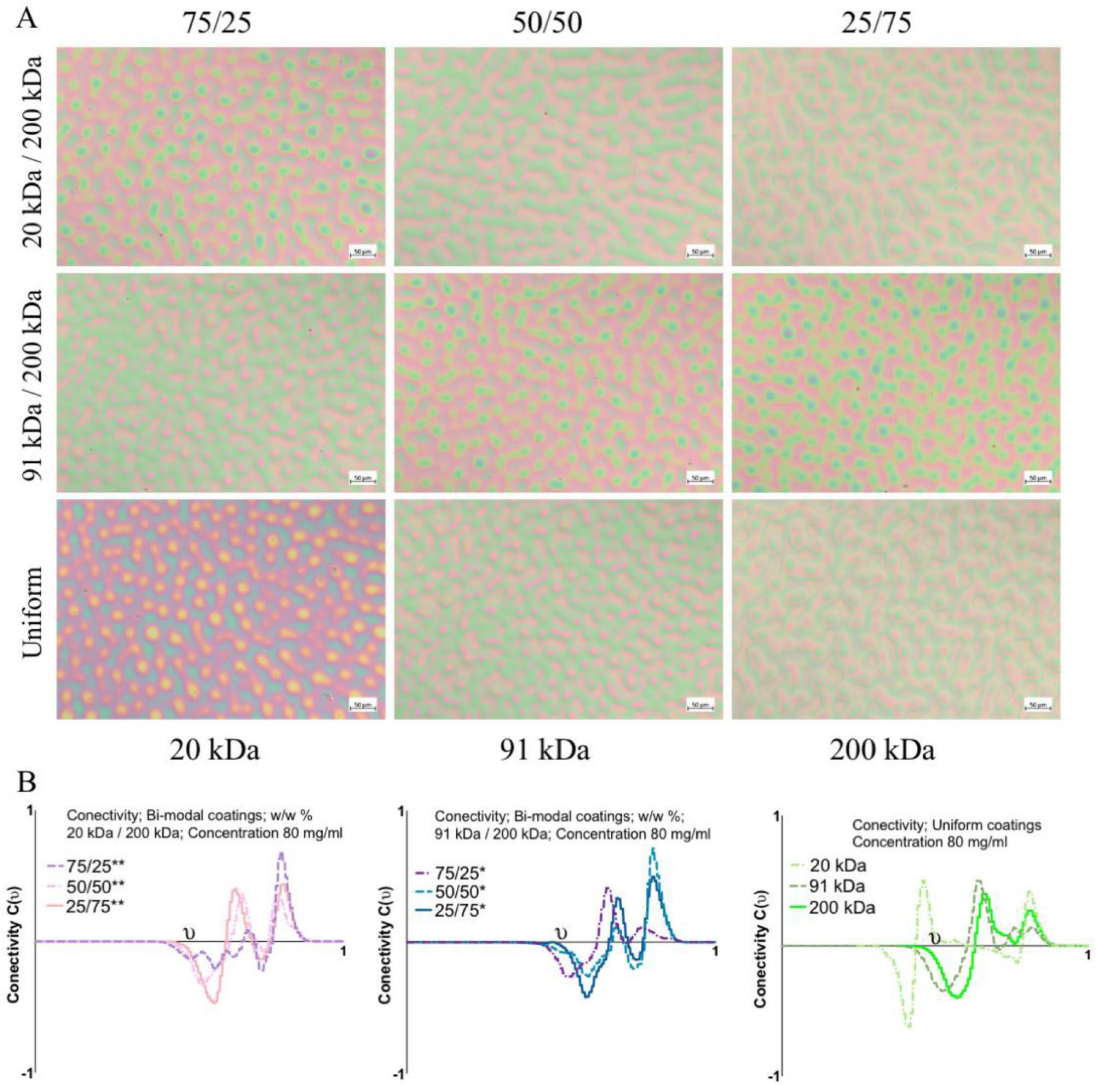
(**A**) Optical images of coatings spun from 80 mg/ml. The (**B**) Minkowski connectivity *C(ν)* of the coatings spun from 80 mg/ml, where ν represents the threshold for image binarization. The values on the axis were normalized to 1.

To represent the morphology of the material quantitatively, one can apply the Minkowski parameters^58^. The images (Fig. 5A) used for analysis come from the central part of the image to exclude the high shear rate effect on the coating morphology. The scale bar was 50 µm. Based on this description, it can be concluded that the morphology of the uniform coatings is characterised by separate islands surrounded by a bicontinuous phase. In contrast, the bimodal coatings are characterised by a bicontinuous phase separated by interconnected islands.

Figure 5B presents connectivity. Connectivity *C*(*ν*) can be used to describe the bicontinuous or island morphology of the coating with respect to the given binarization threshold *ν*. A negative value of connectivity corresponds to bicontinuous morphology, while a positive value corresponds to island morphology. The threshold was normalized to 1. Based on the connectivity, the bimodal coatings had different morphologies than the uniform coatings when the starting conditions (Rh 0%) were considered. With the exclusion of the 75/25 20 kDa 200 kDa coating, the coatings were characterized by peaks related to continuous structures and two peaks related to islands.

In contrast, the uniform coatings had one set of continuous structures and two peaks related to islands. In connection with the images, it can be assumed that these two kinds of islands are one on top of another. In the case of bimodal blends, the presented situation describes a set of interconnected islands on top of another interconnected structure.

### Investigation of coating phase composition by means of AFM force spectroscopy

**Figure 6:**
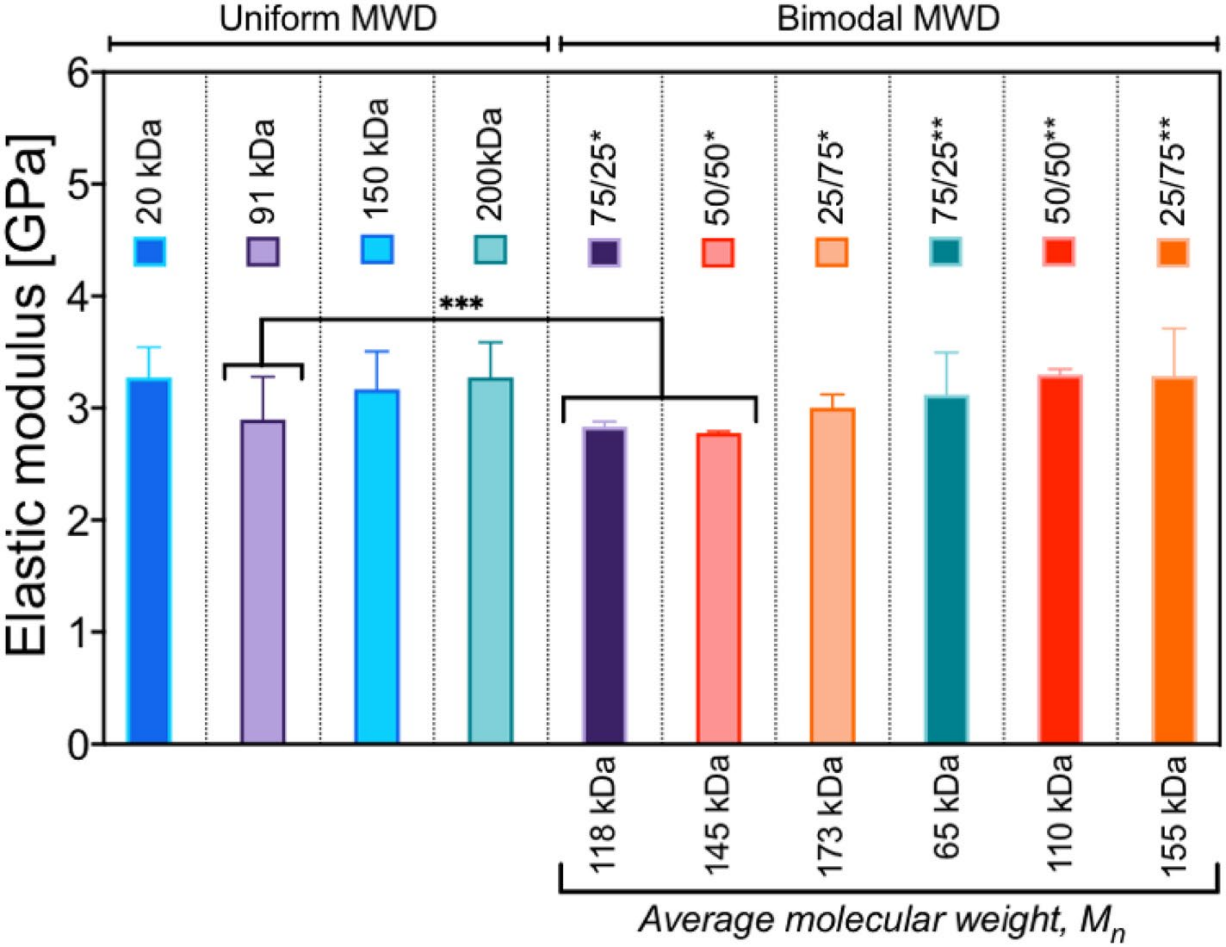
Average elastic modulus obtained based on the FS method for coatings made from a solution of 80 mg/ml. Uniform—coatings were made from homogeneous solutions; bimodal—coatings made from solutions with bimodal MWD; *blends of 91 kDa and 200 kDa polystyrene; **blend of 20 kDa and 200 kDa polystyrene; x/x – w/w% ratio of blended homogeneous polystyrenes. [M_n_] = *f*_*1*_*M*_*w1*_ + *f*_*2*_*M*_*w2*_, where *f* w/w % ratio of polymers. ***means are significantly different (one-way ANOVA, p < 0.05).

The AFM force spectroscopy method allows the visualization and quantification of surface areas differing in mechanical properties^59^. The coatings spun from the solutions with 80 mg/ml concentration were studied. The resulting elastic modulus of the coatings was calculated (Fig. 6).

The obtained results are similar to those found in the literature^60^. The uniform 91 kDa coating and the 75/25 and 50/50 blends of 91 kDa and 200 kDa had significantly lower elastic moduli than the rest of the tested groups. The dependence between the molecular weight and the elastic modulus of the polymer has been repeatedly proven^61,62^. However, we did not find significant differences between the other groups. The uniform 20 kDa coating had an elastic modulus similar to that of the 200 kDa coating in our investigation. The 20 kDa coating was the thinnest; thus, the substrate could influence the result. We performed a linear regression test (SI, *Force Spectroscopy*, Fig. S4) between the thickness of the 80 mg/ml coatings and the elastic modulus, which proved no relationship between the thickness of the coatings and the elastic modulus, while the 20 kDa coating was an outlier (SI, *Force Spectroscopy*, Table S14).

**Figure 7:**
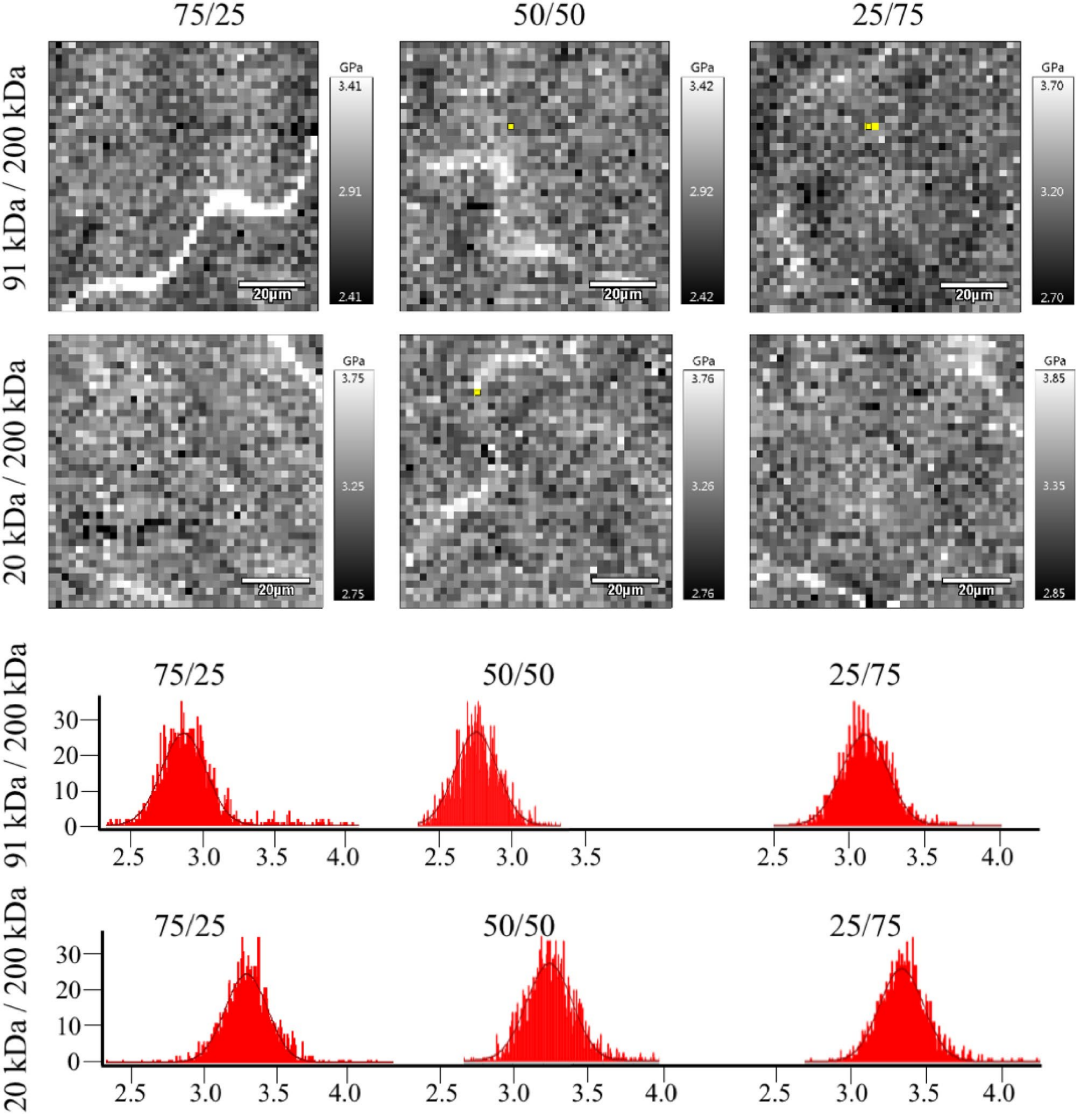
Force spectroscopy maps of bimodal MWD coatings and corresponding histograms of the elastic modulus. The greyscale shows the stiffness—the white colour corresponds to the highest stiffness. The grayscale range is ± 1 GPa.

Interestingly, it was possible to record local differences in the coating surface stiffness (Fig. 7). The maps were gathered for bimodal coatings. White spinodal-like areas are characterized by higher stiffness. The differences are more clearly visible in the case of 90 kDa and 200 kDa blends, in agreement with Fig. 6.

**Figure 8:**
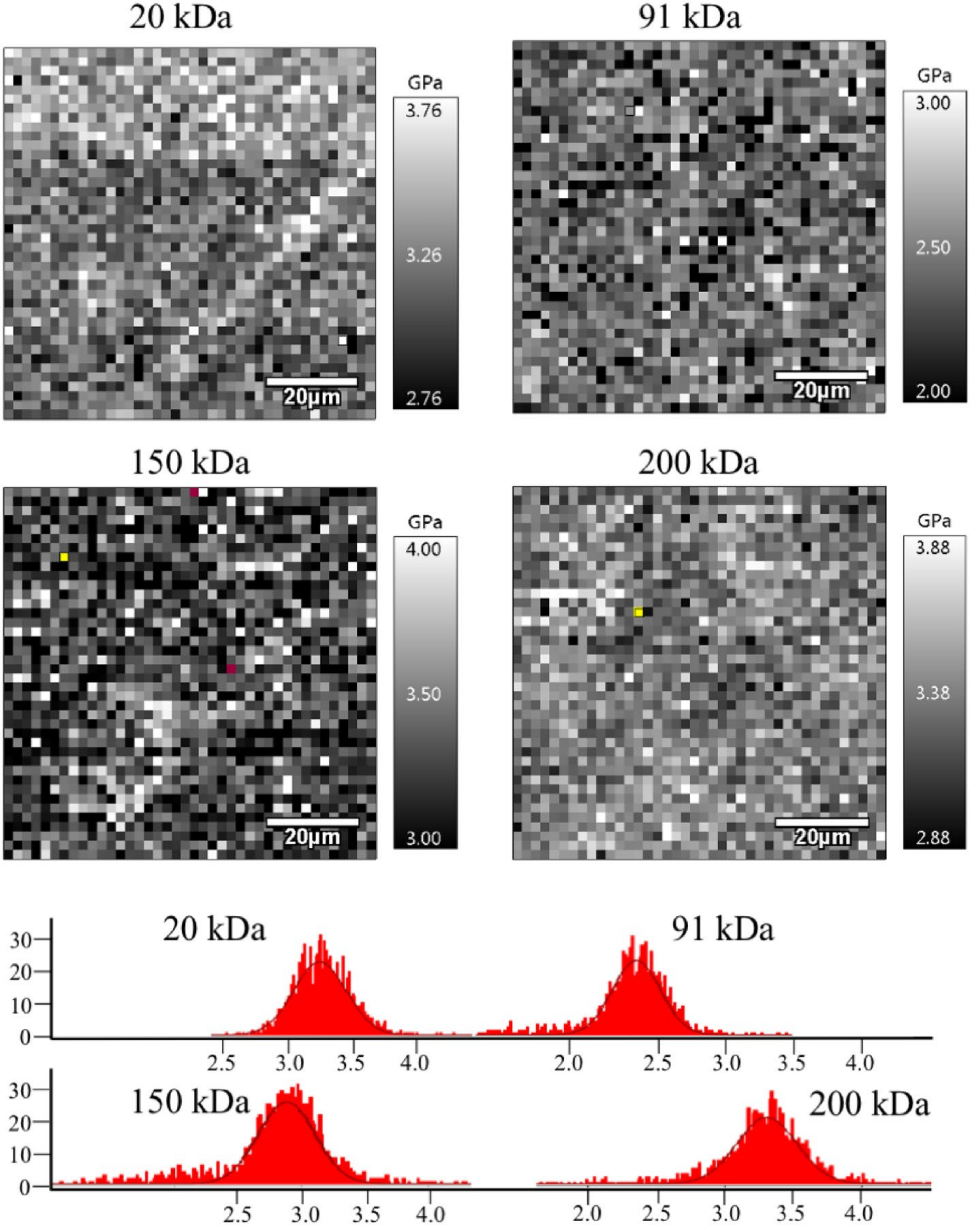
Force spectroscopy maps of coatings with uniform MWD and corresponding histograms of the elastic modulus. The greyscale shows the stiffness—the white colour corresponds to the highest stiffness.

**Figure 9:**
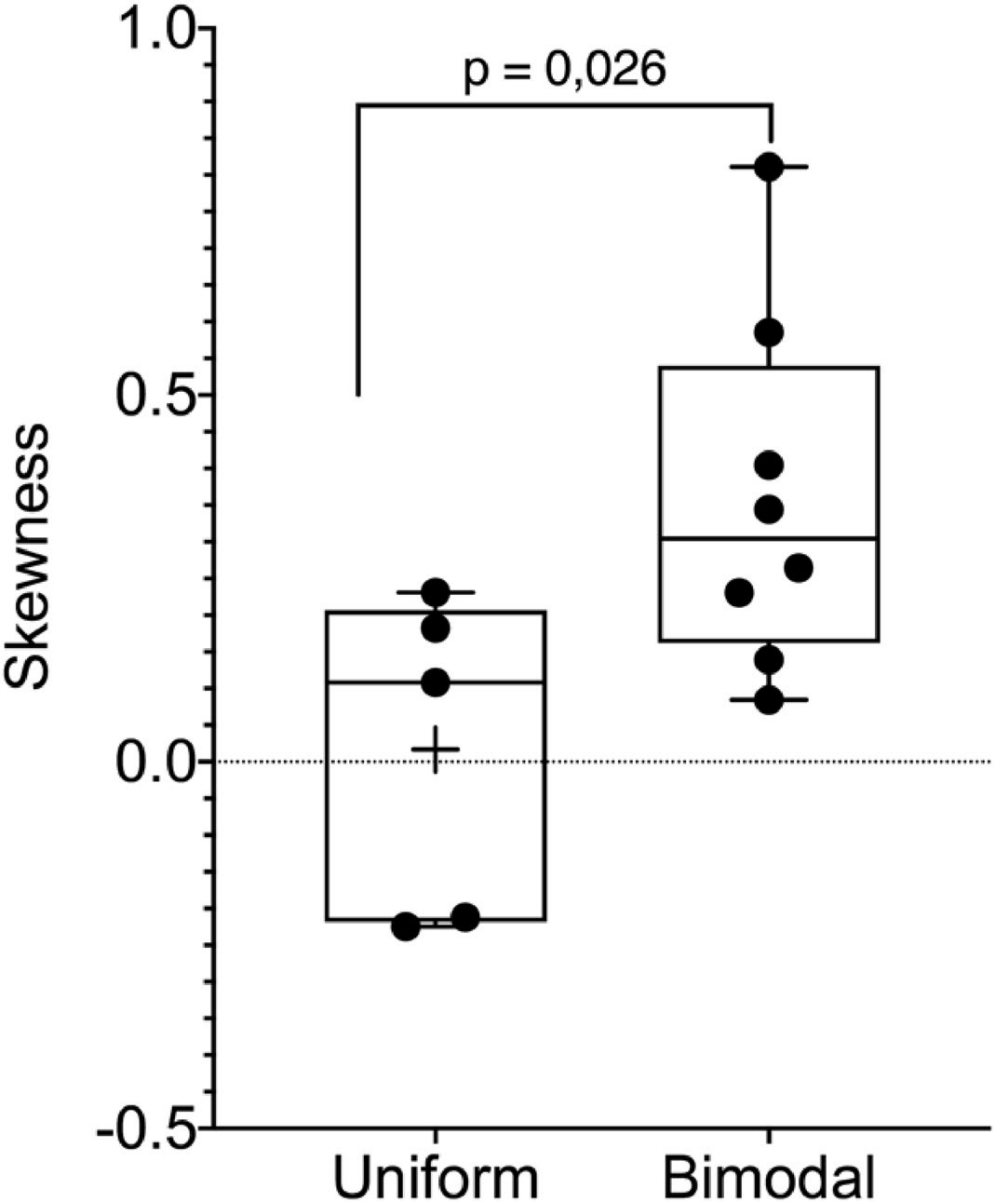
The skewness of the elastic modulus data sets. Uniform—grouped means that represents the skewness of histograms of uniform coatings elastic modulus. Bimodal—grouped means that represents the skewness of histograms of all kinds of bimodal blends elastic modulus. The means of these two groups were significantly different (p < 0.05).

For comparison, Fig. 8 illustrates the FS maps of the uniform coatings. We analysed the skewness of the maps' elastic modulus distribution (SI, *Force Spectroscopy*, Table S15). The skewness in the case of uniform coatings was significantly lower (*p* < 0.05) than that in the case of bimodal coatings (Fig. 9). The distribution of the elastic modulus of the uniform coatings was more homogeneous.

Therefore, it was concluded that force spectroscopy revealed phase separation in the bimodal coatings. The most noteworthy phase separation was found for the 75/25 blends of both kinds of bimodal coatings. Here, the phase of lower concentration formed long, spinodal-like forms. For the 25/75 blends, the separate phases were scattered.

### Solubility of polystyrenes with respect to the molecular weight distribution

The phase separation mentioned above could be explained by solubility investigation. It was shown that the viscosity of the polymeric solution could be utilised by the application of the Mangaraj method to retract several polymer–solvent parameters, i.e. the Flory interaction parameter^63^. We utilised the Mangaraj equation (Eq. 3) to investigate the miscibility gap between the lower- and higher-molecular-weight polystyrenes^64^.

3 $${\text{ln}}\left( {\frac{\eta }{{\eta _{{max}} }}} \right) = - \left( {\delta _{s} - \delta _{{eff}} } \right)^{2}$$

The effective miscibility parameter *δ*_*eff*_ was calculated with respect to a solution of 200 kDa with a concentration of 80 mg/ml, which had the highest viscosity among the tested solutions (*η*_*max*_). The solvent *δ*_*s*_ was set to 19 MPa^0.5^, which is a typical value for MEK.

**Figure 10:**
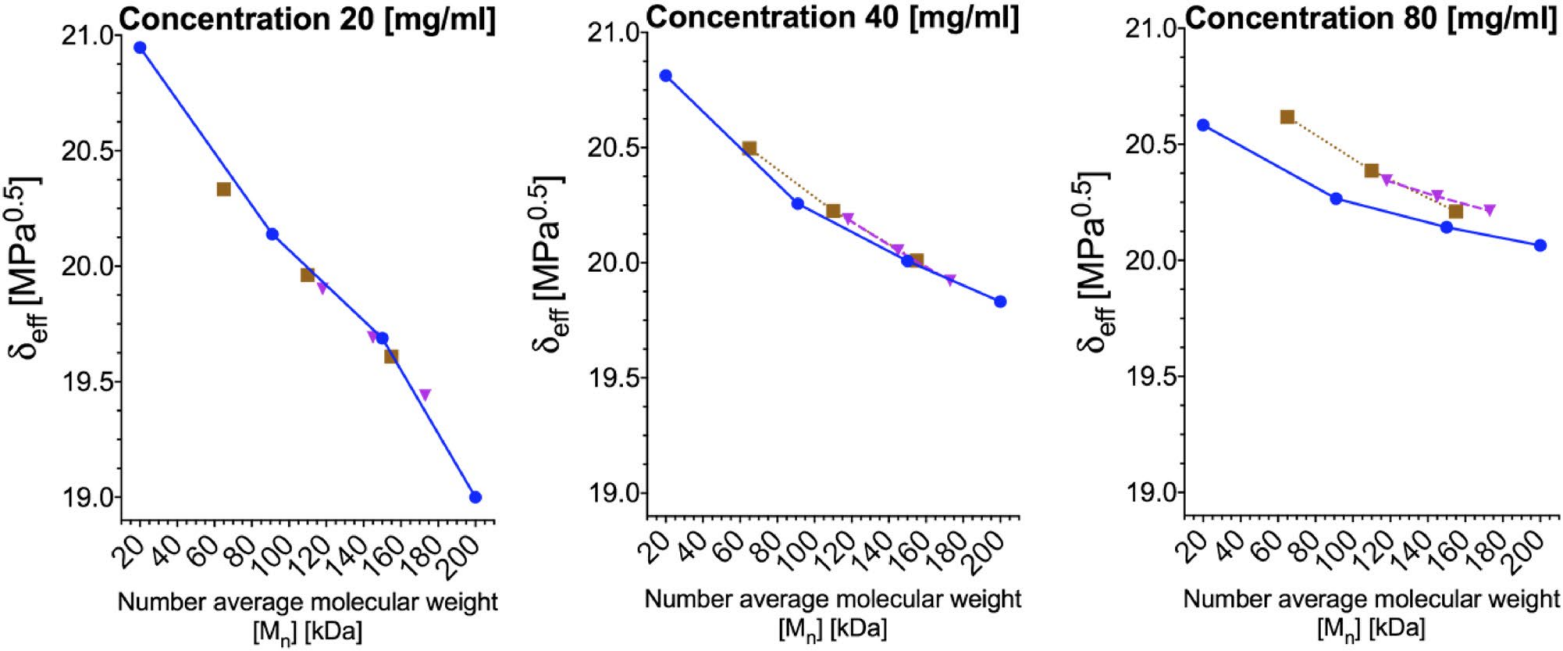
The effective miscibility parameter *δ*_*eff*_ of the polystyrenes in MEK with bimodal and uniform distributions for two kinds of bimodal distributions: 20 kDa/200 kDa—brown squares and 91 kDa/200 kDa—purple triangles; the blue circles represent data for polystyrenes with uniform distributions. [M_n_] = *f*_*1*_*M*_*w1*_ + *f*_*2*_*M*_*w2*_, where *f* w/w % ratio of polymers.

The miscibility gap between low- and high-molecular-weight polystyrene can be derived based on the PS blends' viscosity. Furthermore, the miscibility gap decreases accordingly with the low molecular weight fraction. Here, *δ*_*eff*_ is the effective Hildebrand miscibility parameter calculated based on the intrinsic viscosity *[η]*. For the 20 mg/ml concentration, all the solutions are present on the same linear trend with the lowest *δ*_*eff*_ for the highest molecular weight. It should be noted that with increasing concentration, the trends for 20 kDa/200 kDa solutions (brown squares), 91 kDa/200 kDa solutions (purple triangles), and uniform solutions (blue circles) become divergent at low molecular weights, with their trends coincident at 200 kDa (Fig. 10).

### Self-assembly of micro islands/honeycombs due to phase separation and water condensation

As a follow-up, we utilized humidity to take advantage of the phase separation observed above. It was assumed that phase separation could be further enhanced if spin-coating was performed under humid conditions. It was further concluded that water condensation would eventually lead to ruptures in the solution layer. By this, a new interface between the solution, water, and SiO_2_ substrate would be created. This would induce separation between segregated phases. The condensing water forces the liquid film to dewet and retreat from SiO_2_. The viscosity gradient between different phases would lead to the formation of convection cells and honeycomb morphology^65,66^. It was assumed that the spinodal-like structures found during the FS investigation, probably consisting of a higher molecular weight fraction, would reinforce the honeycomb borders. As revealed by the viscosity investigation, the longer polymer chains would carry the stress as they would be highly entangled. The lower molecular phase would separate because no stress would be applied to the shorter chains. It should also be considered that MEK is a hygroscopic solvent, while PS is slightly hydrophobic. The absorption of water by MEK can further alter the interactions between the solvent and different PS phases. The viscosity and solubility investigations pointed to 80 mg/ml concentration to obtain the most pronounced effect. It was also assumed that water condensation would take place in the later stage of spinning. As the humidity slows evaporation, the highest humidity allows the longest time for morphology formation. The tested relative humidity Rh values were 45%, 55%, and 75%.

**Figure 11:**
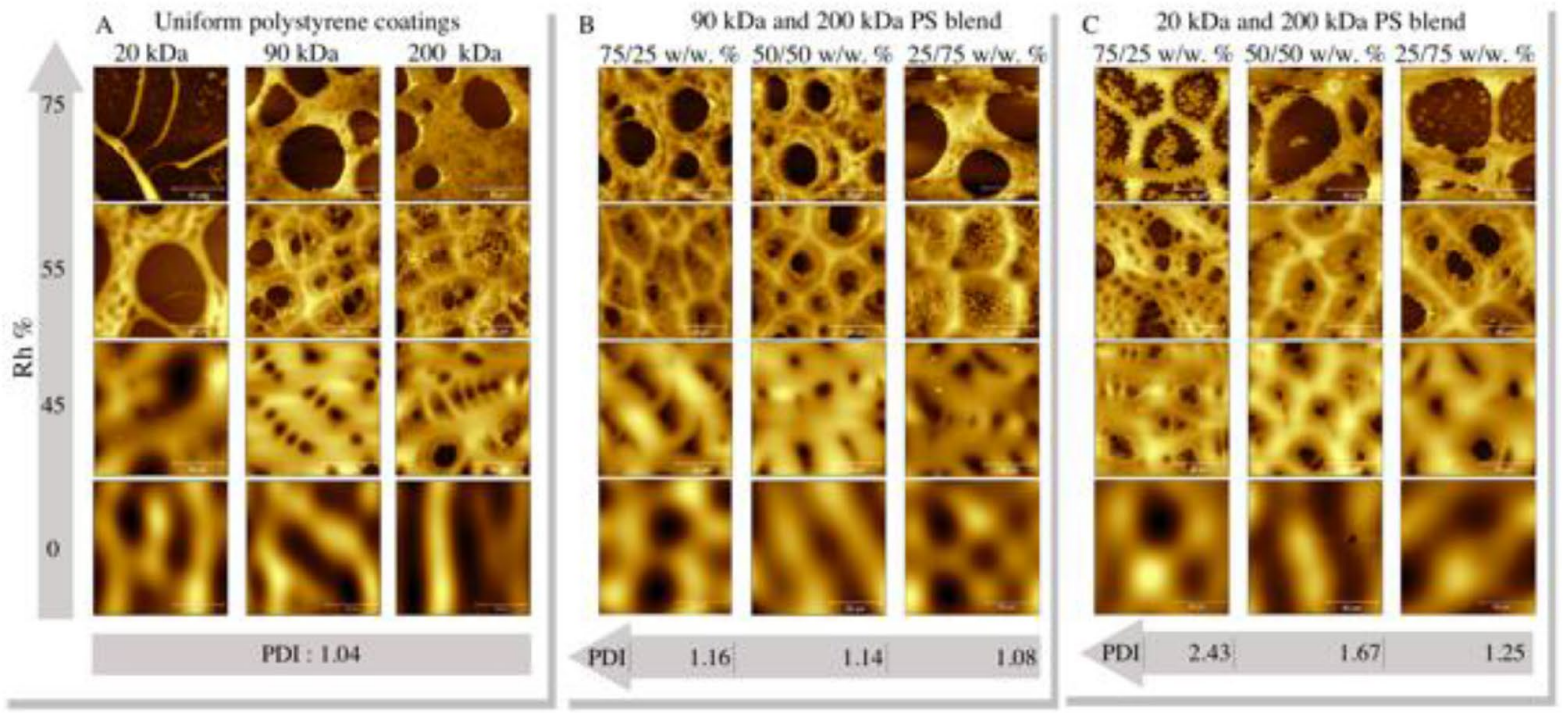
AFM topographical images of tested samples. The colour represents the height, and brighter means higher. The solution concentration C was 80 mg/ml, while the samples were spun under different humidity, from 0 to 75% Rh. The z-scale was chosen for the best representation of the coating morphology. (**A**) uniform coatings; (**B**) 20 kDa and 200 kDa blends; (**C**) 91 kDa and 200 kDa blends.

Figure 11 A shows images of coatings composed of uniform polystyrenes, Fig. 11B presents images of coatings prepared from 91 and 200 kDa blends, and Fig. 11C presents images of coatings prepared from 20 and 200 kDa blends.

Each column is marked below with the mixture's PDI value, and each row corresponds to one of the Rh values. Optical microscopy showing three different areas of the coatings is presented in the Supplementary Information, Figs. S6–S8.

We observed that smaller cavities (breath figures) formed around the larger cavities caused by dewetting-related polymeric film ruptures. The structure that resembles the honeycomb also occurred. For Rh 55%, a honeycomb-like morphology is found for all coatings, except the uniform 20 kDa coating. It is difficult to distinguish uniform samples from the 90 kDa 200 kDa blend. However, the 20 kDa and 200 kDa blends have distinctive morphology that depends on the PDI value. Radially averaged power spectral density analysis (SI, Fig. S9) of four averaged images depicts the differences between the 75/25, 50/50, and 25/75 w/w% 20 kDa and 200 kDa coatings.

**Figure 12:**
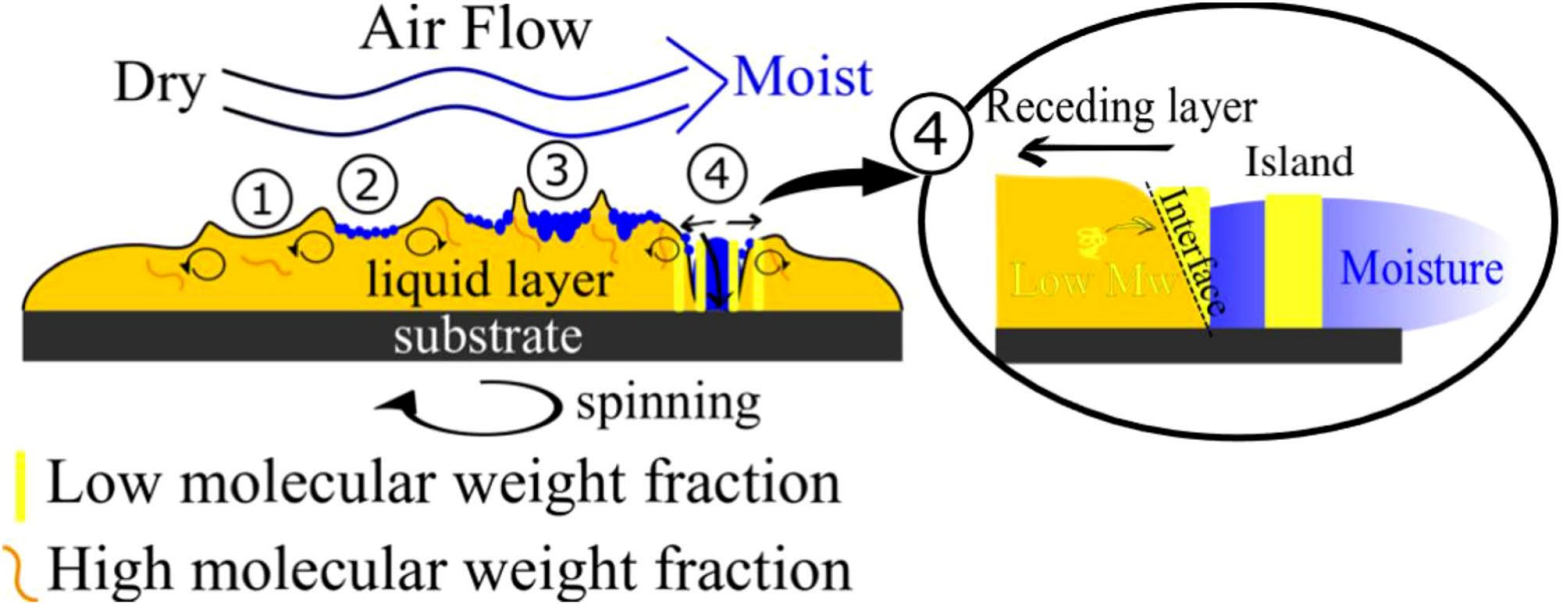
Four scenarios of phase separation: spin-coating in dry air ((1), 0% Rh); moderate humidity ((2), 45% Rh; (3), 55% Rh); and high humidity ((4), 75% Rh). Low and high molecular weight fractions, convective flow driving honeycomb formation, and low molecular weight separation at the interface between the water-wetted rupture and the liquid layer of the polymer solution are presented. (Inkscape v0.92, https://inkscape.org).

Interestingly, the coatings prepared from 20 and 200 kDa bimodal blends at Rh 75% had polymeric islands inside the large holes. The onset of such structures can also be observed in the case of coatings prepared in Rh 55%. The fraction of the islands decreased with an increase in the 200 kDa contribution. It was assumed that the islands are made of a lighter and less viscous fraction. Seemingly, the heavier polymer fraction, more viscous and more entangled, reinforced the honeycomb cell borders. The possible scenario is illustrated in Fig. 12. The coatings at Rh 0%, 45%, 55%, and 75% are marked by numbers 1, 2, 3, and 4, respectively. It is assumed that MEK did not evaporate entirely and formed MEK/20 kDa and MEK/200 kDa fractions. It should be noted that MEK is also soluble in water. Thus, some fraction of the solvent can diffuse to the water phase. The MEK/20 kDa fraction becomes dispersed in the water area of the sample. The MEK/200 kDa fraction forms the walls of the cells. Two explanations can be proposed. First, as shown above, the rheological properties of 200 kDa and 20 kDa polystyrene species differ. The more entangled 200 kDa chains act together, while the 20 kDa chains separate from the high-tension region (higher molecular weight) towards the water/MEK interface. However, water has very high polarity. Very nonpolar polystyrene forms a round shape in contact. Furthermore, as was demonstrated, the solvent would deplete the 20 kDa fraction faster than the 200 kDa fraction, allowing for faster solidification of the 20 kDa islands.

**Figure 13:**
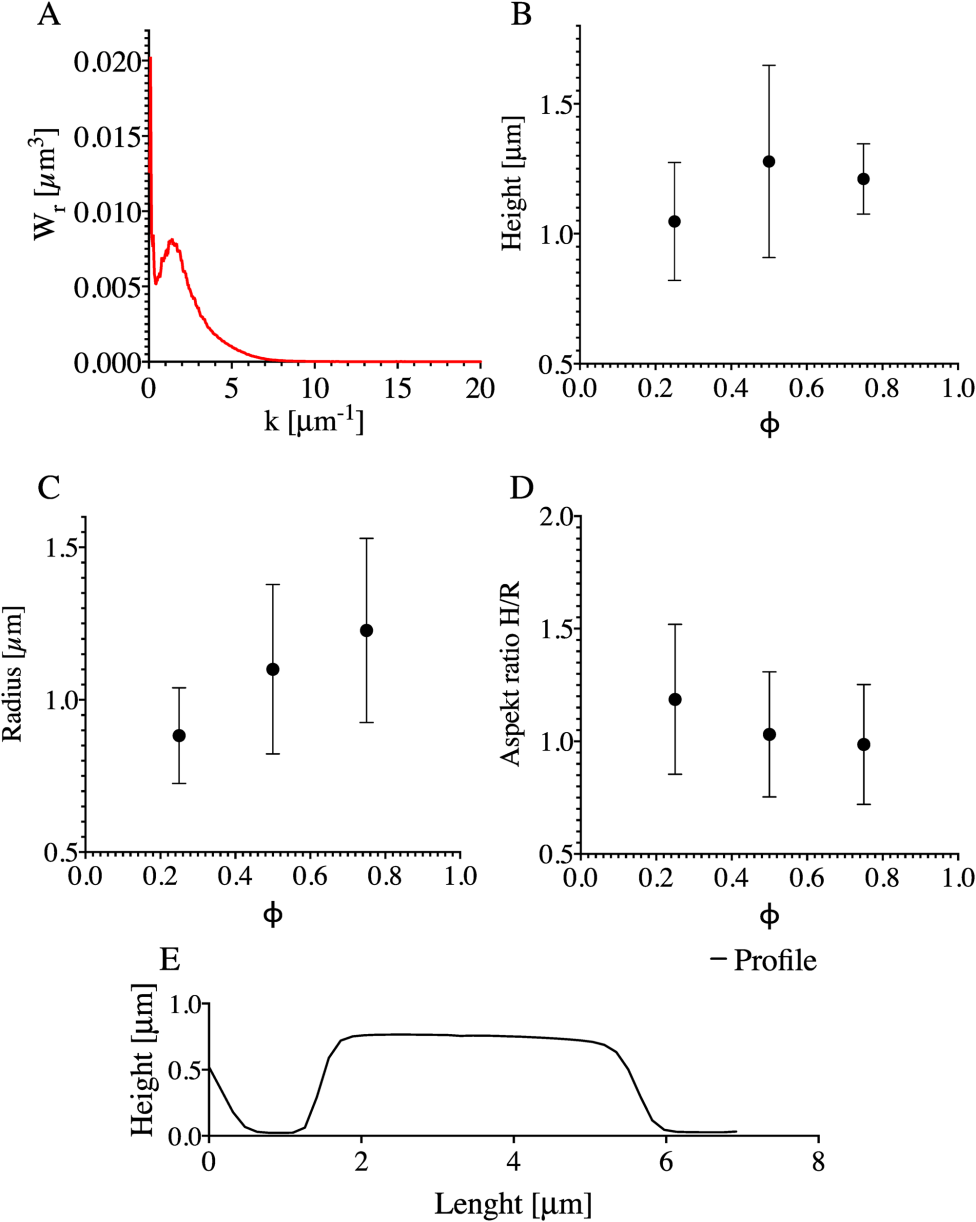
(**A**) Power spectra of islands/honeycomb morphology. (**B**) Mean height, H, of islands with respect to the fraction of 20 kDa PS, ɸ. (**C**) Mean radius of islands, R. (**D**) mean aspect ratio of islands, H/R. H profile of an island.

Second, it can be discussed in terms of the two-step phase separation process discussed by Henderson et al. Their study focused on modelling the two-step quench scenario. First, the solution undergoes a temperature quench into the spinodal decomposition region. After a specific time, a secondary quench was enacted by a further decrease in the temperature. As a result, secondary domains appeared inside the already formed structures^34^. The morphology discussed was very similar to that discussed in the current paper. The presented power spectra of the optical images of the 75/25 w/w% 20 kDa and 200 kDa coatings presented in Fig. 13A coincide with the results presented in the abovementioned paper. Nevertheless, in the current experiment, the first temperature quench is followed by the condensation of water. A decrease in temperature may facilitate the phase separation of polystyrene. Conversely, condensation should coincide with an increase in temperature. ^23^ However, water may be absorbed by MEK. The MEK/water composition would be a much poorer solvent for PS. Thus, secondary quenching would occur due to the change in solvent quality by water.

Figure 13 presents the characteristic values describing the shape of the islands, Fig. 13B–height H, Fig. 13C—radius R, Fig. 13D—aspect ratio H/R and Fig. 13E–profile of an island. The dependence of the shape on the fraction of 20 kDa PS can be pointed out.

Additionally, we tested the formation of micro islands when a short spin-coating time was used (0.5 s and 1 s). These images are presented in Fig. S15 in SI. Secondary phase separation has already been seen, although the islands were not fully formed.

**Figure 14:**
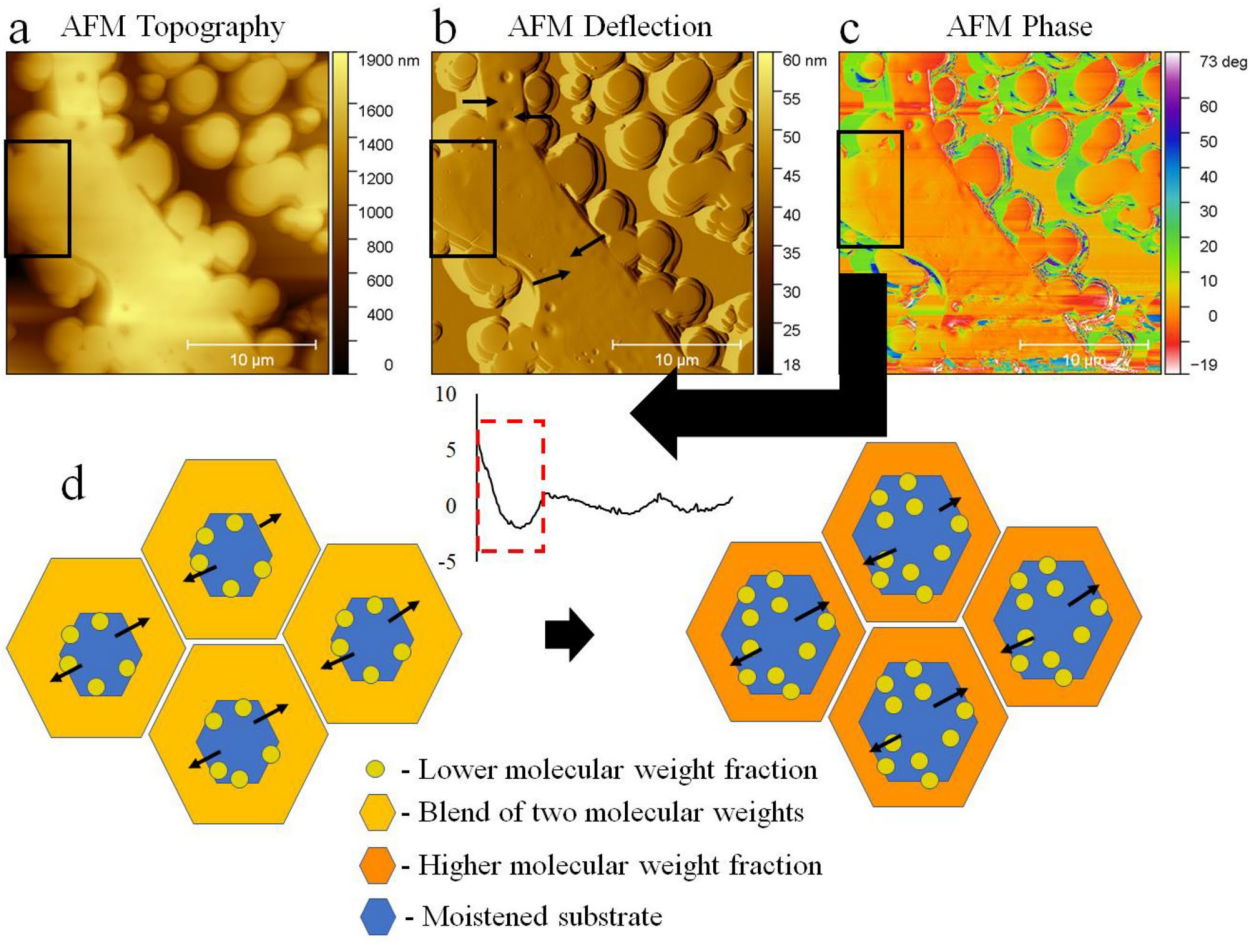
AFM magnification of the cell border of the 20 kDa and 200 kDa 75/25 w/w% blend spun at Rh 75%. The black rectangle indicates the separation of different phases. The same region is shown in the form of (**a**) topography, (**b**) AFM deflection, the direction of dewetting is indicated by the black arrows; (**c**) AFM phase image, an arrow indicates the phase signal difference presented as the averaged cross-section of the marked region (black box); (**d**) schematic illustration depicting proposed formation mechanism of the honeycomb; Convection inside the liquid film led to cells formation, condensation of water led to film rupture (Blue). Subsequently, the recess of the film occurred. At the interface between the rupture area and the receding film, separation of the lower molecular fraction arises (yellow). Subsequently, the cell borders were reinforced by the remaining high molecular weight fraction (Orange).

The AFM image (Fig. 14a–c) shows the event of the low molecular weight fraction separating from the cell border. Cracks between the two phases and the spot where the partially formed island disconnects from the bulk can be seen. The black rectangle indicates the area that differed in terms of the deflection signal and phase contrast. However, the discussed phenomenon appears after the thin film of the solution was formed on the substrate. The condensation required time to make holes in the films. The dewetting of the substrate by the solution can induce additional motion of molecules, allowing the lower molecular weight fraction to precipitate. Water would also change the surface tension locally and induce the thermocapillary effects that lead to the formation of convection cells (depicted in Fig. 14d).

As mentioned, the polystyrenes used were unmodified standard grade polymers. The unchanged chemical composition of the coatings was confirmed by FTIR spectroscopy (SI Fig. S10–S12). The recognized functional groups were CH_3_, CH_2_, and phenyl groups. These groups are hydrophobic and were the driving force behind the dewetting process under high humidity conditions. This argument was further reinforced by the free surface energy (SEF) measurement (SI Figs. S13–S14). The SEF of the 20 kDa coating was slightly higher than that of the 200 kDa coating and, consequently, led to a higher affinity for the hydrophilic SiO_2_ substrate of the former coating.

## Conclusions

To the best of our knowledge, we have demonstrated for the first time phase separation in a solution of polystyrenes with identical chemical structures but different molecular weights. Phase separation was found for 90 kDa and 200 kDa blends and 20 kDa and 200 kDa blends. In both cases, phase separation was found for 75/25 and 50/50 w/w%. Force spectroscopy was used to determine the different phases in the coatings. Phase separation was discussed in terms of the difference in the viscosity of polystyrene species. Next, we provoked secondary phase separation by introducing water vapor. We have theorized that water vapor would act two-fold. The water droplets formed breath figures, and as the vapor concentration increased, they caused raptures in the liquid film. Second, the absorption of vapor by methyl ethyl ketone, a solvent chosen due to its hygroscopic properties, would decrease the solubility of polystyrene. We observed secondary phase separation for the 20 kDa and 200 kDa blends. But not for 90 kDa and 200 kDa blends. We also pointed out that the internal thermocapillary convection and gradient of the surface tension of the top layer formed convection cells inside the liquid. The ruptures caused by condensation, with the combination of convection and thermocapillary effects, formed honeycombs. The secondary phase separation left the dispersed 20 kDa polystyrene phase inside the 200 kDa polystyrene honeycomb.

The described mechanism can likely be applied to other solvent-polymer systems, consisting even of three polymer fractions. It is likely possible to apply the described process to other coating methods, such as dip coating, blade coating or ink-jet printing.

## Experimental

### Materials

All polymers and solvents were purchased from Sigma Aldrich (Merck KGaA). A one-side polished ultrasmooth SiOx wafer was purchased from Technolutions Sp. z o. o.

### Preparation of the coatings

Analytical standard grade polystyrenes (PS) obtained from the supplier with PDI = 1.04 and M_w_ = 20 kDa, 91 kDa, 150 kDa, or 200 kDa were used.

Two kinds of blends were prepared: blends of 20 kDa and 200 kDa PS mixed in 75/25, 50/50, 25/75 w/w % proportions; similarly, blends of 91 kDa and 200 kDa PS were mixed in the same proportions and dissolved in methyl ethyl ketone (analytic grade, MEK) and mixed for an hour at 37 °C. After mixing, the solutions were stored overnight. The list of polystyrene blends used is summarized in Table 1. The concentrations of these solutions ranged from 2.5 to 80 mg/ml. A DIY Arduino-based spin-coater with a chamber with controlled humidity was used to spin polystyrene films onto the SiOx wafers. The experimental setup is depicted in Supplementary Information (SI) Figs. S1–S2. A 35 μl aliquot of the solution was pipetted onto a 1 cm × 1 cm wafer. Spin-coating was performed in a closed chamber with a constant airflow of 10 ml/min to maintain the desired humidity. The rotational speed was 2700 rpm or 3300 rpm. The spinning time was set to 10 s to allow the solvent to evaporate. Solutions were spin-coated under humidity of Rh 0%, 45%, 55%, 75%.

**Table 1:** List of polymer blends that were used for spin-coating.

Type of Blend	Sample code	Molecular weight M_w_ [kDa]	PDI	
Uniform	20 kDa	20	1.04	
	91 kDa	91	1.04	
	150 kDa	150	1.04	
	200 kDa	200	1.04	

Bimodal	Sample code	Molecular weight		PDI
		[M_w_] [kDa] ^a^	[M_n_] [kDa] ^b^	
91 kDa / 200 kDa	75/25*	137	118	1.16
	50/50*	166	146	1.14
	25/75*	186	173	1.08
20 kDa / 200 kDa	75/25**	158	65	2.43
	50/50**	184	110	1.67
	25/75**	194	155	1.25

^a^Weight average molecular weight [M_w_] = (f_1_M_w1_^2^ + f_2_M_w2_^2^)/(f_1_M_w1_ + f_2_M_w2_), ^b^Number average molecular weight [M_n_] = f_1_M_w1_ + f_2_M_w2_, where f—fraction of one of the polymers in %; PDI states for the polydispersity index.

### Gel permeation chromatography (GPC)

The number and weight average molecular weights (M_n_ and M_w_) were determined by an Agilent 1200 series GPC modular system with a refractive index detector (RID) equipped with two PLgel 5 µm MIXED-C columns (300 × 7.5 mm) in the series, while the polydispersity index was calculated as the ratio of M_w_/M_n_. Calibration was performed using a set of 12 narrowly distributed polystyrene standards with molecular weights (Mps) in the range of 474 g/mol—1 800 000 g/mol.

The measurements were performed at 35 °C. Chloroform GPC grade was used as a solvent at a flow rate of 0.7 ml/min. All samples (~ 2 mg/ml) were filtered through a PTFE 0.2 µm membrane before the analysis. The data were collected by ChemStation for LC and analysed by ChemStation GPC Data Analysis Software.

### Force spectroscopy FS and elastic modulus

When a force spectroscopy experiment is performed, an AFM probe applies strain on the film surface^67^. Force spectroscopy was performed by means of atomic force microscopy (AFM, Asylum Research MFP3D Bio)^68^. An OMLCT-AC200TS-R3 (Olympus) cantilever was used with the nominal spring constant k = 9 N/m and tip radius below 10 nm, as suggested by the cantilever’s producer. AFM was calibrated using the built-in thermal vibrations method. The^69^ Johnson, Kendall, and Roberts (JKR) model was used to calculate the elastic modulus (E)^70^. The indentation depth was ~ 8 nm (Supplementary Information, *4. Force Spectroscopy*). As we wanted to neglect the possible influence of stiff (silica) substrate on the polystyrene coating's registered mechanical data, we decided to perform an FS experiment using the thickest films (films spun from the solution of a concentration of 80 mg/ml).

Maps of a large area of the coating 80 μm × 80 μm with 40 × 40 points were obtained. These higher resolution maps are supplemented as an attachment (SI). These data were supported by lower resolution maps with a resolution of 15 × 15 points. Each map was used to obtain the mean elastic modulus value. Altogether, at least five maps were made. Ordinary one-way ANOVA followed by multiple comparisons Fisher’s test was used to compare different groups' means.

Furthermore, histograms representing each higher resolution map were prepared. The skewness of the elastic modulus distribution was measured. Skewness was divided into two groups: one for uniform coatings and one for bimodal coatings. The t-test (*p* < 0.05) was used to compare the means of these two groups.

### Evaluation of the thickness of spin-coated films by means of atomic force microscopy

The thickness of spin-coated films was assessed based on intentionally made scratch topography images (SI Fig. S3). Five randomly selected areas of each sample were tested, and profiles were generated. Each of the profile lines was averaged from three contiguous lines to avoid any unwanted artefacts.

### Imaging of the coatings

Inverted light microscopes (Nikon EPIPHOT 200 and Zeiss Axio Observer) were used for imaging. An atomic force microscope (AFM, Asylum Research MFP3D Bio) working in tapping mode (AC mode) was used to illustrate the phase composition and topography of the polystyrene films.

### In situ measurement of evaporation during spin-coating through laser light reflectometry with stroboscopic effect

In situ stroboscopic laser light reflectometry was developed to investigate the thinning of the solution layer while spinning. The laser light is reflected from the coating during the spin-coating process. The occurring interference pattern can be used to estimate the thinning rate of the solution^71–73^. The experimental setup is described in SI, Figs. S1–S2.

Depending on the thickness of the layer, constructive or destructive interference can occur. The condition for the constructive interference was calculated from Bragg’s law: $2n\Delta hcos\theta =m\lambda$, where *n* is the refractive index of the layer, *Δh* is the thickness of the layer, *θ* is the incident angle, *m* is an integer number, and *λ* is the light wavelength. For pure MEK, *Δh* = 235 nm (assuming the refractive index MEK = 1.3788). For the polymer solution, it was assumed that the refractive index was *n* = 1.5; thus, *Δh* = 217 nm. The laser light wavelength was *λ* = 650 nm. The time resolution was 0.022 s.

### Data analysis and visualisation

For data visualization and analysis: Microsoft PowerPoint 365, Inkscape v0.92, and GraphPad Prism was used. Image analysis procedures implemented in Gwyddion software (ver 2.50) and Igor Pro 6.37 with Asylum Research 15.02.105 add-on were used. Spectragryph v1.5.15 was used for FTIR analysis.

### Fourier transform IR

Infrared spectra were collected using a Fourier transform infrared spectrophotometer (Nicolet 8700 FTIR, Thermo Scientific). Measurements were performed using FTIR ATR over a range of 4000–400 cm^−1^.

### Contact angle and surface free energy measurement

The contact angle (CA) was measured using a Data Physics OCA 20 goniometer. The contact angle was measured with a sessile drop method. For surface free energy measurement (SFE), two kinds of coatings were chosen: 20 kDa and 200 kDa. For each type of coating, three droplets were measured, and three different coatings were used. Two liquid systems were used: deionised water and diiodomethane (Sigma Aldrich, Analytic grade). The groups were compared with the t-test (*p* < 0.05). The Owens, Wendt, Rabel, and Kaelble (OWKR) method was used for SFE calculation^74^.

## Supporting Information (SI)

Experimental setup depicting the custom-built spin-coater with a humidity-controlled chamber and an in situ interferometer with the stroboscopic effect. Illustration of thickness measurement. Viscosity-related coefficients. Optical microscope images. FTIR results. Free Surface Energy results.

## Supplementary Information

Supplementary Information.

## Abbreviations

AFM: Atomic force microscopy
Bimodal: Polymer with two nodes in molecular weight distribution
Coating: Final polymer coating
CA: Contact angle
E: Elastic modulus
Film: Liquid film of solution spread on the substrate
FS: Force spectroscopy
FTIR: Fourier transform IR
GPC: Gel permeation chromatography
Initial solution: Solution at the start of spin-coating
MEK: Methyl ethyl ketone
Mw: Molecular weight
[M_w_]: Weight average molecular weight
[M_n_]: Number average molecular weight
MWD: Molecular weight distribution
PDI: Polydispersity index
PTF: Polymer thin film
PS: Polystyrene
Rh%: Relative humidity in %
RMS: Root mean square roughness
Uniform: Polymer with one node in molecular weight distribution

## References

[1] Kumar M, Bhardwaj R (2020). Wetting characteristics of *Colocasia esculenta* (Taro) leaf and a bioinspired surface thereof. Sci. Rep..

[2] He R, Xiao J, Zhang M, Zhang Z, Zhang W, Cao Y (2016). Artificial honeycomb-inspired TiO_2_ nanorod arrays with tunable nano/micro interfaces for improving poly(dimethylsiloxane) surface hydrophobicity. J. Mater. Sci..

[3] Jahed Z, Shahsavan H, Verma MS, Rogowski JL, Seo BB, Zhao B (2017). Bacterial networks on hydrophobic micropillars. ACS Nano.

[4] Liu X, Liu R, Cao B, Ye K, Li S, Gu Y (2016). Subcellular cell geometry on micropillars regulates stem cell differentiation. Biomaterials.

[5] Walheim S, Böltau M, Mlynek J, Krausch G, Steiner U (1997). Structure formation via polymer demixing in spin-cast films. Macromolecules.

[6] Daly R, Sader JE, Boland JJ (2013). The dominant role of the solvent–water interface in water droplet templating of polymers. Soft Matter.

[7] Khikhlovskyi V, Wang R, van Breemen AJJM, Gelinck GH, Janssen RAJ, Kemerink M (2014). Nanoscale organic ferroelectric resistive switches. J. Phys. Chem. C.

[8] D’Andrade BW, Forrest SR (2004). White organic light-emitting devices for solid-state lighting. Adv. Mater..

[9] Yabu H (2018). Fabrication of honeycomb films by the breath figure technique and their applications. Sci. Technol. Adv. Mater..

[10] Wu D, Xu F, Sun B, Fu R, He H, Matyjaszewski K (2012). Design and preparation of porous polymers. Chem. Rev..

[11] Karagkiozaki V, Vavoulidis E, Karagiannidis PG, Gioti M, Fatouros DG, Vizirianakis IS (2012). Development of a nanoporous and multilayer drug-delivery platform for medical implants. Int. J. Nanomed..

[12] Calejo MT, Ilmarinen T, Skottman H, Kellomäki M (2018). Breath figures in tissue engineering and drug delivery: state-of-the-art and future perspectives. Acta Biomater..

[13] Vendra, V. K., Wu, L., & Krishnan, S. Polymer Thin Films for Biomedical Applications. In: Nanotechnologies for the Life Sciences. Weinheim: Wiley-VCH Verlag GmbH & Co. KGaA (2011). 10.1002/9783527610419.ntls0179.

[14] Griesser, H. J. *Thin Film Coatings for Biomaterials and Biomedical Applications*, 1st Edition, p. 310 (2016).

[15] Łojkowski M, Walheim S, Jokubauskas P, Schimmel T, Święszkowski W (2019). Tuning the wettability of a thin polymer film by gradually changing the geometry of nanoscale pore edges. Langmuir.

[16] Plawsky JL, Kim JK, Schubert EF (2009). Engineered nanoporous and nanostructured films. Mater Today.

[17] Bormashenko E (2017). Breath-figure self-assembly, a versatile method of manufacturing membranes and porous structures: physical, chemical and technological aspects. Membranes (Basel).

[18] Birnie DP (2001). Rational solvent selection strategies to combat striation formation during spin coating of thin films. J. Mater. Res..

[19] van Franeker JJ, Westhoff D, Turbiez M, Wienk MM, Schmidt V, Janssen RAJ (2015). Controlling the dominant length scale of liquid-liquid phase separation in spin-coated organic semiconductor films. Adv. Funct. Mater..

[20] Schaefer C, Michels JJ, van der Schoot P (2016). Structuring of thin-film polymer mixtures upon solvent evaporation. Macromolecules.

[21] Ebbens S, Hodgkinson R, Parnell AJ, Dunbar A, Martin SJ, Topham PD (2011). In situ imaging and height reconstruction of phase separation processes in polymer blends during spin coating. ACS Nano.

[22] Danglad-Flores J, Eickelmann S, Riegler H (2018). Deposition of polymer films by spin casting: a quantitative analysis. Chem. Eng. Sci..

[23] Dombrovsky LA, Frenkel M, Legchenkova I, Bormashenko E (2020). Effect of thermal properties of a substrate on formation of self-arranged surface structures on evaporated polymer films. Int. J. Heat Mass Transf..

[24] Daly R, Sader JE, Boland JJ (2016). Taming self-organization dynamics to dramatically control porous architectures. ACS Nano.

[25] Zabusky HH, Heitmiller RF (1964). Properties of high density polyethylenes with bimodal molecular weight distribution. Polym Eng Sci..

[26] Wu BH, Zhong QZ, Xu ZK, Wan LS (2017). Effects of molecular weight distribution on the self-assembly of end-functionalized polystyrenes. Polym. Chem..

[27] Jiang H, Zhang L, Qin J, Zhang W, Cheng Z, Zhu X (2012). Producing bimodal molecular weight distribution polymers through facile one-pot/one-step RAFT polymerization. J. Polym. Sci. Part A Polym. Chem..

[28] Whitfield R, Parkatzidis K, Truong NP, Junkers T, Anastasaki A (2020). Tailoring polymer dispersity by RAFT polymerization: a versatile approach. Chem..

[29] Tanaka K, Takahara A, Kajiyama T (1997). Effect of polydispersity on surface molecular motion of polystyrene films. Macromolecules.

[30] Heitmiller RF, Naar RZ, Zabusky HH (1964). Effect of homogeneity on viscosity in capillary extrusion of polyethylene. J. Appl. Polym. Sci..

[31] Koningsveld R, Chermin HAG, Gordon M (1970). Liquid─liquid phase separation in multicomponent polymer solutions-VIII. Stability limits and consolute states in quasi-ternary mixtures. Proc. R. Soc. Lond. A Math. Phys. Sci..

[32] Zeman L, Patterson D (1972). Effect of the solvent on polymer incompatibility in solution. Macromolecules.

[33] Shultz AR, Flory PJ (1952). Phase equilibria in polymer—solvent systems 1,2. J. Am. Chem. Soc..

[34] Henderson IC, Clarke N (2004). Two-step phase separation in polymer blends. Macromolecules.

[35] Tanaka H (2012). Viscoelastic phase separation in soft matter and foods. Faraday Discuss..

[36] Hengeller L, Huang Q, Dorokhin A, Alvarez NJ, Almdal K, Hassager O (2016). Stress relaxation of bi-disperse polystyrene melts: exploring the interactions between long and short chains in non-linear rheology. Rheol. Acta.

[37] Harris EK (1973). Effect of blending on the rheological properties of polystyrene. J. Appl. Polym. Sci..

[38] Klein J (1978). The onset of entangled behavior in semidilute and concentrated polymer solutions. Macromolecules.

[39] Hong KM, Noolandi J (1981). Theory of inhomogeneous multicomponent polymer systems. Macromolecules.

[40] Hariharan A, Kumar SK, Russell TP (1990). A lattice model for the surface segregation of polymer chains due to molecular weight effects. Macromolecules.

[41] Mahmoudi P, Matsen MW (2017). Entropic segregation of short polymers to the surface of a polydisperse melt. Eur. Phys. J. E..

[42] Hill JA, Endres KJ, Mahmoudi P, Matsen MW, Wesdemiotis C, Foster MD (2018). Detection of surface enrichment driven by molecular weight disparity in virtually monodisperse polymers. ACS Macro Lett..

[43] Stein GE, Laws TS, Verduzco R (2019). Tailoring the attraction of polymers toward surfaces. Macromolecules.

[44] Carlier V, Sclavons M, Jonas AM, Jérôme R, Legras R (2001). Probing thermoplastic matrix−carbon fiber interphases 1. Preferential segregation of low molar mass chains to the interface. Macromolecules.

[45] Suwa J, Kakiage M, Yamanobe T, Komoto T, Uehara H (2007). Molecular weight segregation on surfaces of polyethylene blended films as estimated from nanoscratch tests using scanning probe microscopy. Langmuir.

[46] Karim A, Slawecki TM, Kumar SK, Douglas JF, Satija SK, Han CC (1998). Phase-separation-induced surface patterns in thin polymer blend films. Macromolecules.

[47] Hoppe H, Heuberger M, Klein J (2001). Self-similarity and pattern selection in the roughening of binary liquid films. Phys. Rev. Lett..

[48] Heier J, Kramer EJ, Revesz P, Battistig G, Bates FS (1999). Spinodal decomposition in a subsurface layer of a polymer blend film. Macromolecules.

[49] Jandt KD, Heier J, Bates FS, Kramer EJ (1996). Transient surface roughening of thin films of phase separating polymer mixtures. Langmuir.

[50] Huang C, Förste A, Walheim S, Schimmel T (2015). Polymer blend lithography for metal films: large-area patterning with over 1 billion holes/inch2. Beilstein. J. Nanotechnol..

[51] Flory PJ, Höcker H (1971). Thermodynamics of polystyrene solutions. Part 1.—Polystyrene and methyl ethyl ketone. Trans. Faraday Soc..

[52] Imre A, Van Hook WA (1996). Liquid-liquid demixing from solutions of polystyrene. 1. A review. 2. Improved correlation with solvent properties. J. Phys. Chem. Ref. Data.

[53] Utracki, L. A., & Wilkie, C. A. *Polymer Blends Handbook*, pp. 1–2378 (2014).

[54] Ying Q, Chu B (1987). Overlap concentration of macromolecules in solution. Macromolecules.

[55] Kim JK, Taki K, Nagamine S, Ohshima M (2008). Periodic porous stripe patterning in a polymer blend film induced by phase separation during spin-casting. Langmuir.

[56] Heier J, Kramer EJ, Groenewold J, Fredrickson GH (2000). Kinetics of individual block copolymer island formation and disappearance near an absorbing boundary. Macromolecules.

[57] Coveney S, Clarke N (2014). Pattern formation in polymer blend thin films: surface roughening couples to phase separation. Phys. Rev. Lett..

[58] Mecke K (2000). Additivity, convexity, and beyond: applications of minkowski functionals in statistical physics. Stat. Phys. Spat. Stat..

[59] Wolf BA (1997). Improvement of polymer solubility: influence of shear and of pressure. Pure Appl. Chem..

[60] Du B, Tsui OKC, Zhang Q, He T (2001). Study of elastic modulus and yield strength of polymer thin films using atomic force microscopy. Langmuir.

[61] Landel RF, Nielsen LE (1993). Mechanical Properties of Polymers and Composites.

[62] Torres JM, Stafford CM, Vogt BD (2010). Impact of molecular mass on the elastic modulus of thin polystyrene films. Polymer (Guildf)..

[63] Kok CM, Rudin A (1982). Prediction of Flory–Huggins interaction parameters from intrinsic viscosities. J. Appl. Polym. Sci..

[64] Lee SH, Lee SB (2005). The Hildebrand solubility parameters, cohesive energy densities and internal energies of 1-alkyl-3-methylimidazolium-based room temperature ionic liquids. Chem. Commun..

[65] Bormashenko E, Malkin A, Musin A, Bormashenko Y, Whyman G, Litvak N (2008). Mesoscopic patterning in evaporated polymer solutions: Poly(ethylene glycol) and room-temperature-vulcanized polyorganosilanes/-siloxanes promote formation of honeycomb structures. Macromol. Chem. Phys..

[66] Uchiyama H, Matsui T, Kozuka H (2015). Spontaneous pattern formation induced by bénard–marangoni convection for sol–gel-derived titania dip-coating films: effect of co-solvents with a high surface tension and low volatility. Langmuir.

[67] Zgłobicka I, Chlanda A, Woźniak M, Łojkowski M, Szoszkiewicz R, Mazurkiewicz-Pawlicka M (2017). Microstructure and nanomechanical properties of single stalks from diatom Didymosphenia geminata and their change due to adsorption of selected metal ions. J. Phycol..

[68] Chlanda A, Kijeńska-Gawrońska E, Zdunek J, Swieszkowski W (2020). Internal nanocrystalline structure and stiffness alterations of electrospun polycaprolactone-based mats after six months of in vitro degradation. An atomic force microscopy assay. J. Mech. Behav. Biomed. Mater..

[69] Sader JE, Borgani R, Gibson CT, Haviland DB, Michael J, Kilpatrick JI (2016). A virtual instrument to standardise the calibration of atomic force microscope cantilevers. Rev Sci Instrum..

[70] Wu KC, You HI (2007). Determination of solid material elastic modulus and surface energy based on JKR contact model. Appl. Surf. Sci..

[71] Toolan DTW (2015). Straightforward technique for in situ imaging of spin-coated thin films. Opt. Eng..

[72] Toolan DTW, Howse JR (2013). Development of in situ studies of spin coated polymer films. J. Mater. Chem. C.

[73] Mokarian-Tabari P, Geoghegan M, Howse JR, Heriot SY, Thompson RL, Jones RAL (2010). Quantitative evaluation of evaporation rate during spin-coating of polymer blend films: control of film structure through defined-atmosphere solvent-casting. Eur. Phys. J. E.

[74] Drelich JW, Boinovich L, Chibowski E, Della VC, Hołysz L, Marmur A (2019). Contact angles: history of over 200 years of open questions. Surf. Innov..

