# Supplementary material for: Water﻿ vapor induced self-assembly of islands/honeycomb structure by secondary phase separation in polystyrene solution with bimodal molecular weight distribution
Source: Sci Rep. 2021 Jun 24;11:13299. doi: 10.1038/s41598-021-92594-1 (PMC8225630; doi:10.1038/s41598-021-92594-1)
Supplement: Supplementary file 1 — Supplementary Information. [file 41598_2021_92594_MOESM1_ESM.docx]

# *Supporting information.*

**Water vapor induced self-assembly of islands/honeycomb structure by secondary phase separation in polystyrene solution with bimodal molecular weight distribution**

Authors: Maciej Łojkowski^1,^*, Adrian Chlanda^1,2^, Emilia Choińska^1^, Wojciech Swieszkowski^1^*.

Affiliation:

1. Faculty of Material Sciences and Engineering, Warsaw University of Technology, Wołoska 141, 02-507 Warsaw, Poland
2. Department of Chemical Synthesis and Flake Graphene, Institute of Electronic Materials Technology, Wólczyńska 133, 01-919 Warsaw, Poland

*Corresponding authors:

Maciej Łojkowski*, ORCID: 0000-0002-0612-7964, email: [00183042@pw.edu.pl](mailto:00183042@pw.edu.pl)

Wojciech Swieszkowski*, ORCID: 0000-0003-4216-9974, email: wojciech.swieszkowski@pw.edu.pl.

| Number of Pages | 31 |
| --- | --- |
| Number of Figures | 15 |
| Number of Tables | 16 |

**Table of content**

1. **Experimental setup description**

Fig. S1. DIY spin-coater with humidity controller and laser reflectometer.

Fig. S2. Evaporation rate by laser reflectometry.

1. **Film thickness analysis.**

Fig. S3 Illustration of thickness measurement

1. **Viscosity analysis**

Table S1. K and [η] coefficients, 95% confidence interval CI; goodness of the fit.

Table S2. Coefficients for Mark-Houwink equation.

Table S3. Dynamic viscosity values of the uniform solutions in cP.

1. **Force Spectroscopy analysis**

Table S4. Blend of 20 kDa and 200 kDa polystyrene; 75/25 w/w%; Rh 0%.

Table S5. Blend of 20 kDa and 200 kDa polystyrene; 50/50 w/w%; Rh 0%.

Table S6. Blend of 20 kDa and 200 kDa polystyrene; 25/75 w/w%; Rh 0%.

Table S7. Blend of 91 kDa and 200 kDa polystyrene; 75/25 w/w%; Rh 0%.

Table S8. Blend of 91 kDa and 200 kDa polystyrene; 50/50 w/w%; Rh 0%.

Table S9. Blend of 91 kDa and 200 kDa polystyrene; 25/75 w/w%; Rh 0%.

Table S10. 20 kDa uniform polystyrene standard; Rh 0%.

Table S11. 91 kDa uniform polystyrene standard; Rh 0%.

Table S12. 150 kDa uniform polystyrene standard; Rh 0%.

Table S13. 200 kDa uniform polystyrene standard; Rh 0%.

Table S14. Mean elastic modulus in GPa.

Table S15. Skewness of the elastic modulus histograms.

Fig. S4. Regression test between the thickness of the coatings and the elastic modulus.

Table S16. Are the slops of the linear regression between the thickness of the coatings and the elastic modulus significantly different than zero?

1. **Optical imaging**

Fig. S5 Example photographies of wafers coated with polystyrene films.

Fig. S6**.** Optical microscope images of the coatings prepared from the uniform solutions 20 kDa, 90 kDa and 200 kDa or the bimodal solutions: 75/25, 50/50 or 25/75 w/w %; humidity 45% or 55%.

Fig. S7. Optical microscope images of the coatings prepared from the uniform solutions with molecular weight of polystyrene: 20 kDa, 90 kDa and 200 kDa, respectively; humidity Rh 75%.

Fig. S8. Optical microscope images of the bimodal coatings prepared from 20 kDa and 200 kDa blends, humidity 75%, molecular weight ratio w/w: 75/25, 50/50 and 25/75, respectively.

Fig. S9. Radially averaged Powers Spectra Density of coatings images, comparison between coatings made from different ratios of 20 kDa and 200 kDa polystyrene at humidity Rh 75%. Spectra were made by averaging 4 images.

1. **Fourier Transform IR spectroscopy**

Fig. S10. Fourier Transform IR spectra of 90 kDa and 200 kDa coatings on SiO_2_. Compositions 75/25, 50/50 and 25/75 w/w % were investigated.

Fig. S11. Fourier Transform IR spectra of 20 kDa and 200 kDa coatings on SiO_2_. Compositions 75/25, 50/50 and 25/75 w/w % were investigated.

Fig. S12. FTIR spectra of SiO_2_ wafer.

1. **Free Surface Energy and contact angle**

Fig. S13. Contact angle on 20 kDa and 200 kDa uniform coatings.

Fig. S14. Free Surface Energy of 20 kDa and 200 kDa coatings. Polar and dispersive components.

1. **Varying the spin-coating time**

Fig. S15. PS 20 kDa and 200 kDa blend, 75/25 ratio, Spin-coating time: 0.5s and 1s

1.
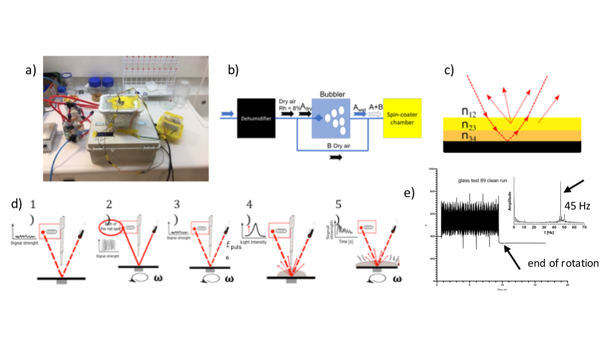
 **Experimental setup the custom build spin-coater with humidity-controlled chamber and in-situ reflectometer utilizing stroboscopic effect.**

Fig S1. a) DIY spin-coater with humidity controller; b) Scheme of the humidity controller; c) illustration of the path of the light passing through multiple layers, part of the light is being scattered, n_12,_ n_23,_ n_34_ - the refractive index; d) illustration of the stroboscopic effect on filtering out the undesirable signal; e) unfiltered signal and its Fast Fourier Transform, the frequency is exact 45 Hz (equal to 2700 rpm); (Image processing software: Inkscape v0.92, <https://inkscape.org>; GraphPad Prism 9, https://www.graphpad.com/scientific-software/prism/)

Fig. S1 illustrates the costume setup used for the experiment is depicted in Fig. S1 a) shows picture of the experimental setup. The spin-coater, was build according to Ref. [[1]]. A custom Arduino code was written to control the spin-coater. Fig. S1 b) shows the humidity controller. The ambient air flows into the dehumidifier. The dry air flow is split, part of it flows through the bubbler to take up moisture. Regulation of the ratio between the dry and the moist air allows to sustain the desirable humidity. The mixed wet and dry air flows into the spin-coater chamber. During presented experiment, only dry air was allowed to flow into the chamber. The humidity sensor inside the chamber measured the relative humidity (Rh). The sensor indicated the relative humidity Rh = 0%.

The system built for the investigation of the evaporation of the solvent during the spin-coating consisted of the Arduino microcontroller, the red laser diode (𝛌= 650nm, 5mW) and light dependent resistor (LDR, Velleman VMA407). A custom Arduino code was written. The resistance of the LDR changes with the intensity of light that interacts with the resistor. The higher is light intensity the higher is the registered voltage.

The angle of incident of the laser light, taken from the device geomery, was around 5°. Thus, for calculations the incident beam will be treated as normal to the surface. Fig. S1c) illustrates light passing through the multiple layers that have got diferent refractive indexes n_ij_. The refractive index n of the solution can be calculated from:

$n_{mix}=\frac{m_{p}n_{p}+m_{s}n_{s}}{m_{p}+m_{s}}$_,_

where m_i_ and n_i_ is molecular mas and refractive index of polymer and solvent respectively.[2]. For the MEK/PS system the refractive index can be assumed as n = 1.4, while for the MEK the refractive index is n = 1.38 and for PS the refractive index is n = 1.5.

The position of the laser diode, the LDR, sample and the pipet with the solution is depicted in Fig. S1 d) 2) illustrates that the sample wobles during the rottation. In result, the graph presenting the signal shows fringes with frequency corresponding with the rotational speed of the sample rotation. To overcame this technical problem, the Arduino was programed to utilize the stroboscopic effect. The laser diode and LDR were turned on and off with frequency correspoonding with the rotational speed. Fig. S1 d) 3) shows that the applied algoritm filters the unwanted signal. Fig. S1 d) 4) shows that, when the solution is pipeted onto the wafer, the laser light is scattered and the signal readeing increases. Fig. S1 d) 4) shows that when the thickness of the layer of the spin-coated solution is decreasing, fringes occurs in the LDR reading in the function of time. The fringes are due to the interference between light reflected from the top of the layer and the bottom of the layer.

In Fig. S1 e) graph presents actual signal reading during spinning the clean SiO wafer, without the filtering with the stroboscope algoritm and the Fast Fouruer Transform (FFT) of that signal. The main peak The main peak at 45 Hz shows that the spin-coater was properly calibrated and the rotation speed was in fact 2700 rpm.

For the test of the in-situ evaporation measurement set of solvents was used. In Fig. S2 a) 35 µl of methyl ethyl ketone (MEK) was dropped on the wafer and spun with frequency of 45 Hz (2700 rpm). The LDR voltage amplitude is presented as a function of time. Fig. S2 b) presents the measured voltage amplitude, when the clean wafer was rotating. As it can be seen, the fringes from the wobbling of the sample, seen in Fig. S1 e), were filtered out. Small amplitude jump is seen, it marks the start of the rotation, the spin-coater needs time to accelerate. During acceleration the device and the spin-coater are out of phase. This period is short and lasts around 0.2 s.

The curve showing the evaporating of MEK as seen in Fig. S2 a) consist of two regions. First, during which interference fringes can be seen, lasts 0.92 s. The second phase lasts until 5.89 s and depicts the end of the MEK film evaporation. This is with agreement with results found in the literature. [3] The first phase is often depicted as the “hydrodynamic thinning”, while the following phase is referred as “solvent thinning” phase.

The difference in length of the light path reflected from the top and bottom of the optical layer result in the interference between these two beams of light. The condition for minimum and maximum of the interreference fringes can be taken from Bragg’s equation: 2nd cosθ = mλ, where n is the refractive index of the layer, d is thickness of the layer, θ – incident angle, m is integer and λ is wavelength. The distance between two interference maximums is Δd = 235 nm (refractive index of MEK n_MEK_=1.3788). In case of solution of PS in MEK the refractive index is assumed to be 1.5 and the Δd is 217 nm. The interference fringes can occur only if the layer is thinning and its thickness is more than Δd. If the thickness of the layer drops below the Δd, the next fringes would not appear. Moreover, as the film becomes thinner the amount of scattered light decreases as I_s_ ̴ I_0_exp[-d], it can be assumed that the signal output will decrease with time of the spin-coating.

The rate of evaporation of the solution during spin-coating can be defined as the difference between first and last interference fringes.

The total evaporation rate R = Δt / V_solution_.

And the thinning rate of the film created by the spin-coated solution is:

The thinning rate dh/dt = Δd / Δt.


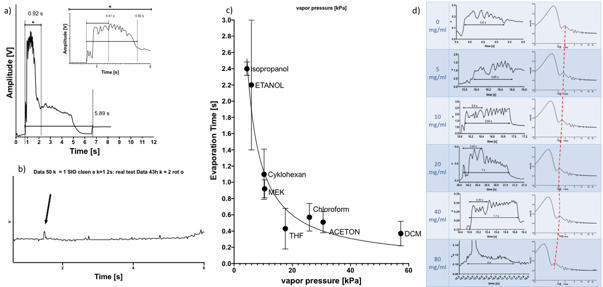


Fig. S2 a) Intensity of the light measured as a LDR voltage [V] in function of time [s] during spin-coating 35 µl of MEK; b) reference signal obtained during the rotation of the clean SiOx wafer; c) graph presents the evaporation times for different test solvents; d) graphs obtained for the increasing concentration of PS in MEK, and the corresponding Fast Fourier Transforms of the signals; (Image processing software: PowerPoint 365, https://office.live.com; GraphPad Prism 9, https://www.graphpad.com/scientific-software/prism/)

Fig. S2 c) shows the evaporation time between first and last fringe for different solvents plotted as function of their vapor pressure. The fitting curve is power series in form of A*X^B+C*X^D.

As can be seen, the evaporation rate of solvent with simmilar vapor pressure are simmilar, i.e. cyclohexane and MEK.

Fig. S2 d) shows the evaporation curves for the mixture of polystyrene with MEK in different concentrations c. Corresponding Fast Fourier Transforms (FFT) of the signals are shown. The second frequency corresponds to the duration of the fringes. The progressive shift towards the lower frequencies shows increasing duration of the fringes in respect to the polymer concentration. Thus, the rate of the evaporation can be retrieved from the FFT analysis.

1. **Film thickness analysis.**

**
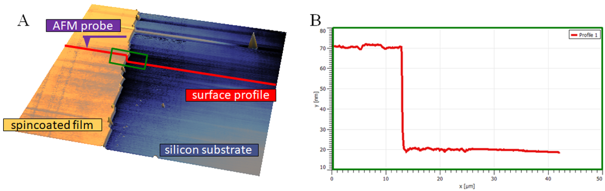
**

Fig S3. A) Schematic illustration of spin-coated film’s thickness assessment, B) Representative topographical profile a sample recorded using AFM. (Image processing software: PowerPoint 365, https://office.live.com; Gwyddion v2.50, http://gwyddion.net/)

1. **Viscosity analysis**

Table S1. Coefficients K and [η] for relation between viscosity and concentration, $\frac{\eta_{r}}{c}=[\eta](1+K\left[ \eta\right]c+\frac{(K\left[ \eta\right]{c)}^{2}}{2}+\frac{\left( K\left[ \eta\right]c \right)^{3}}{6})$; 95% confidence interval CI; goodness of the fit coefficients: R^2^ and sum of squares SS.

|  | [η] | K | 95% CI | | Fit quality | |
| --- | --- | --- | --- | --- | --- | --- |
|  |  |  | [η] | K | R^2^ | SS |
| Uniform 20 | 0.012 | 0.33 | 0.006 to 0.020 | -0.66 to 2.71 | 0.022 | 0.0027 |
| Uniform 91 | 0.048 | 0.10 | 0.037 to 0.060 | -0.01 to 0.26 | 0.215 | 0.0032 |
| Uniform 150 | 0.035 | 0.40 | 0.025 to 0.046 | 0.21 to 0.76 | 0.758 | 0.0029 |
| Uniform 200 | 0.052 | 0.36 | 0.046 to 0.058 | 0.29 to 0.46 | 0.975 | 0.0017 |
| 75/25** | 0.013 | 1.01 | 0.011 to 0.015 | 0.70 to 1.46 | 0.922 | 0.0001 |
| 50/50** | 0.029 | 0.40 | 0.026 to 0.031 | 0.32 to 0.49 | 0.934 | 0.0004 |
| 25/75** | 0.026 | 0.93 | 0.017 to 0.035 | 0.55 to 1.77 | 0.919 | 0.0028 |
| 75/25* | 0.018 | 1.36 | 0.013 to 0.023 | 0.89 to 2.25 | 0.940 | 0.0014 |
| 50/50* | 0.033 | 0.54 | 0.030 to 0.036 | 0.46 to 0.63 | 0.986 | 0.0003 |
| 25/75* | 0.035 | 0.61 | 0.029 to 0.040 | 0.47 to 0.81 | 0.973 | 0.0020 |

** - Blends of 91 kDa and 200 kDa polystyrene; ** - blend of 20 kDa and 200 kDa polystyrene; x/x – w/w% ratio of blended uniform polystyrenes.*

Table S2. Coefficients for Mark-Houwink equation.

|  | c 80 [mg/ml] | c 40 [mg/ml] | c 20 [mg/ml] | c 10 [mg/ml] |
| --- | --- | --- | --- | --- |
| Uniform | | | | |
| K | -10.29 | -8.23 | -5.86 | -5.72 |
| CI 95% | -13,28 to -8,03 | -12,30 to -5,84 | -9,69 to -3,84 | -7,04 to -4,75 |
| a | 1.65 | 1.13 | 0.60 | 0.55 |
| CI 95% | 1,21 to 2,22 | 0,66 to 1,91 | 0,18 to 1,35 | 0,35 to 0,81 |
| R^2^ | 0,953 | 0,895 | 0,510 | 0,841 |
| SS | 0,004 | 0,002 | 0,005 | 0,001 |
| Bimodal | | | | |
| K | -10.09 | -9.99 | -7.53 | -11.16 |
| CI 95% | -10,97 to -9,26 | -11,22 to -8,88 | -8,80 to -6,35 | -18,89 to -5,67 |
| a | 1.62 | 1.47 | 0.87 | 1.58 |
| CI 95% | 1,45 to 1,79 | 1,25 to 1,72 | 0,631 to 1,13 | 0,463 to 3,11 |
| R^2^ | 0,959 | 0,948 | 0,729 | 0,388 |
| SS | 0,002 | 0,001 | 0,001 | 0,004 |

*CI - Confidence Interval; SS - sum of squares.*

Table S3. Dynamic viscosity values of the uniform solutions in cP. Viscosity of Pure MEK: 0.39 ± 0.04 cP.

| 1. Uniform | | | | | | | | |
| --- | --- | --- | --- | --- | --- | --- | --- | --- |
| Concentration [mg/ml] | PS 20 kDa | | PS 90 kDa | | PS 150 kDa | | PS 200 kDa | |
| 5 | 0.39 ± 0.04 | | 0.49 ± 0.05 | | 0.48 ± 0.04 | | 0.50 ± 0.05 | |
| 10 | 0.39 ± 0.04 | | 0.55 ± 0.05 | | 0.58 ± 0.03 | | 0.63 ± 0.03 | |
| 20 | 0.45 ± 0.01 | | 0.85 ± 0.11 | | 0.64 ± 0.10 | | 0.99 ± 0.07 | |
| 40 | 0.59 ± 0.02 | | 1.22 ± 0.04 | | 1.34 ± 0.24 | | 2.15 ± 0.07 | |
| 80 | 0.95 ± 0.02 | | 2.60 ± 0.55 | | 3.73 ± 0.24 | | 7.18 ± 0.25 | |
| 1. Bimodal | | | | | | | | |
| Concentration [mg/ml] | 20 kDa and 200 kDa blend | | | | 90 kDa and 200 kDa blend | | | |
|  | 75/25 | 50/50 | | 25/75 | 75/25 | 50/50 | | 25/75 |
| 5 | 0.42 ± 0.01 | 0.46 ± 0.02 | | 0.43 ± 0.02 | 0.43 ± 0.01 | 0.46 ± 0.01 | | 0.44 ± 0.01 |
| 10 | 0.44 ± 0.01 | 0.53 ± 0.02 | | 0.57 ± 0.10 | 0.43 ± 0.01 | 0.57 ± 0.02 | | 0.55 ± 0.05 |
| 20 | 0.53 ± 0.01 | 0.66 ± 0.03 | | 0.66 ± 0.02 | 0.65 ± 0.05 | 0.75 ± 0.03 | | 0.78 ± 0.01 |
| 40 | 0.78 ± 0.02 | 1.09 ± 0.05 | | 1.47 ± 0.03 | 1.24 ± 0.05 | 1.39 ± 0.05 | | 1.88 ± 0.02 |
| 80 | 1.52 ± 0.03 | 2.63 ± 0.12 | | 5.14 ± 0.32 | 3.72 ± 0.26 | 4.45 ± 0.04 | | - 1. 0,15 |

**4. Force Spectroscopy**

The blends were prepared by spin coating the polystyrene blend solution on a SiOx wafer. Force maps were prepared using AFM force spectroscopy. Each point on the map represents the value of the elastic modulus at that point. Humidity during spinning was kept close to 0%. All force maps are scaled to the same range as follows: mean ± 1 GPa. All elastic modulus histogram bins were scaled to the same scale.

Table S4. Blend of 20 kDa and 200 kDa polystyrene; 75/25 w/w%; Rh 0%. (Image processing software: Igor Pro 6.37 with Asylum Research 15.02.105 add-on, https://afm.oxinst.com)

| 1. | |
| --- | --- |
| 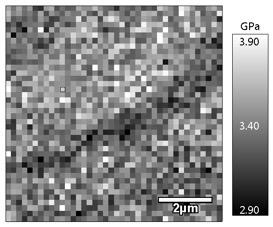 | Elastic modulus histogram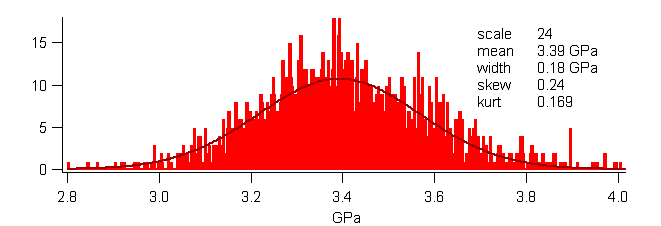    Histogram of indentation depth  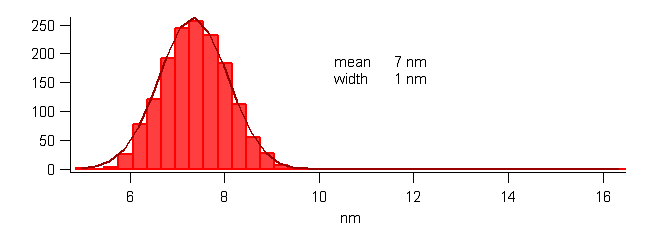 |
| 2. | |
| 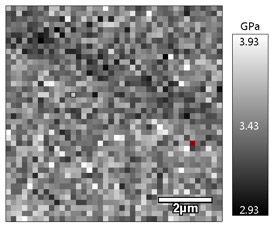 | Elastic modulus histogram 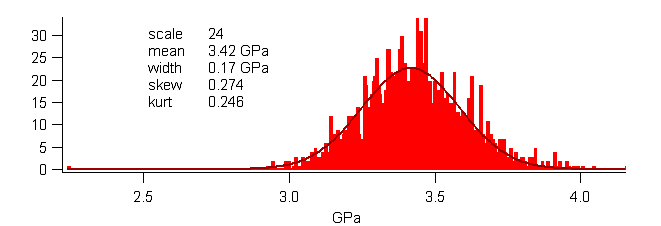  Histogram of indentation depth  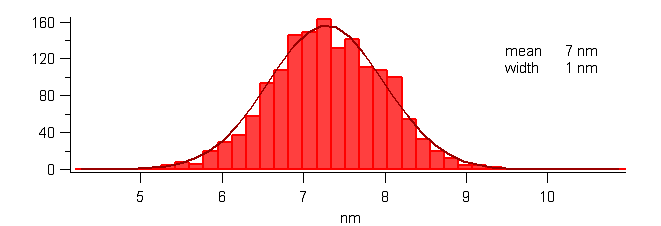 |

| 3. | |
| --- | --- |
| 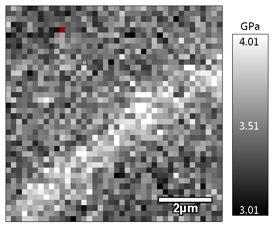 | Elastic modulus histogram 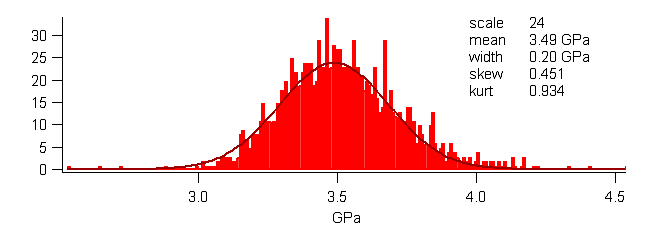 |
| 4. | |
| 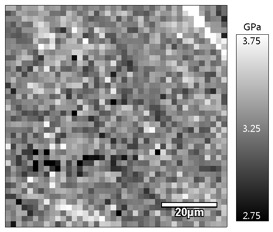 | Elastic modulus histogram 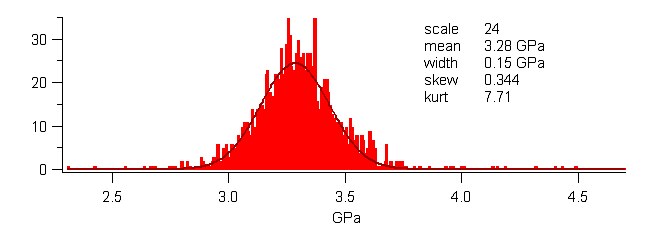  Indentation depth 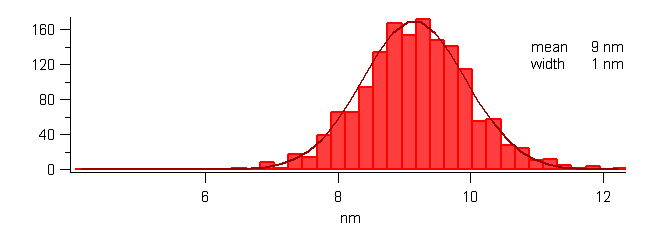 |
| 5. | |
| 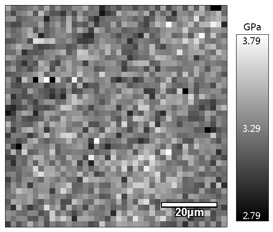 | Elastic modulus histogram 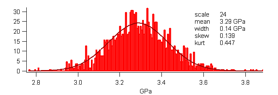 |

Table S5. Blend of 20 kDa and 200 kDa polystyrene; 50/50 w/w%; Rh 0%.(Image processing software: Igor Pro 6.37 with Asylum Research 15.02.105 add-on, https://afm.oxinst.com)

| 1. | |
| --- | --- |
| 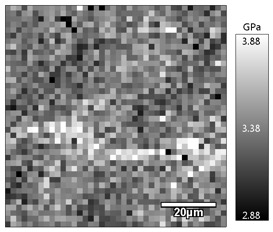 | Elastic modulus histogram 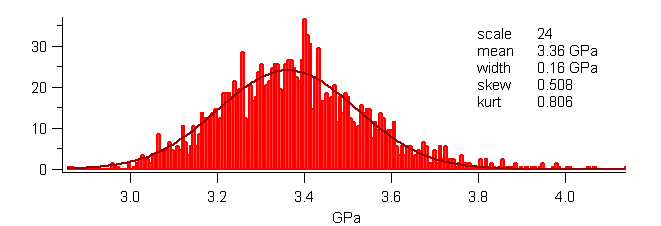 |
| 2. | |
| 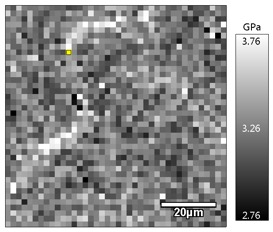 | Elastic modulus histogram 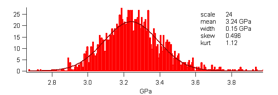  Indentation depth 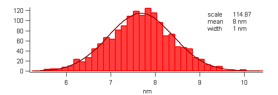 |

Table S6. Blend of 20 kDa and 200 kDa polystyrene; 25/75 w/w%; Rh 0%.

| 1. | |
| --- | --- |
| 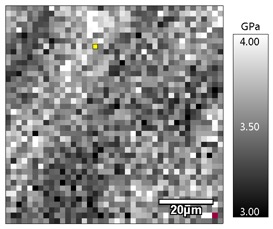 | Elastic modulus histogram 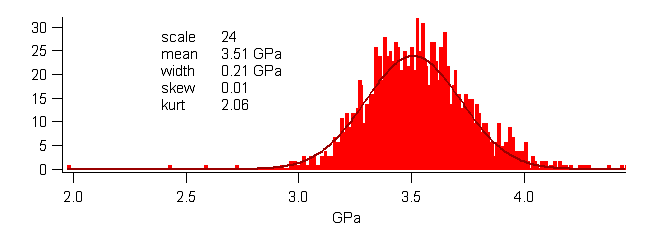 |

| 2. | |
| --- | --- |
| 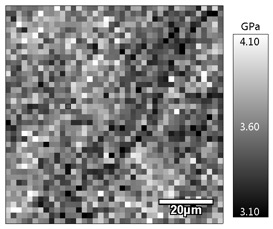 | Elastic modulus histogram 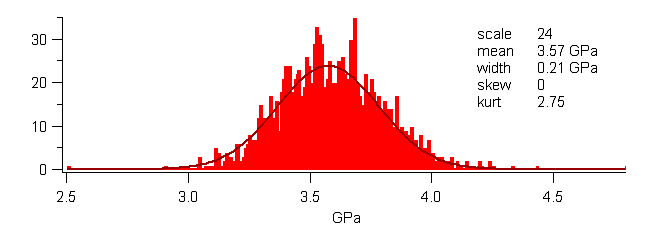  Indentation depth 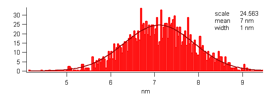 |
| 3. | |
| 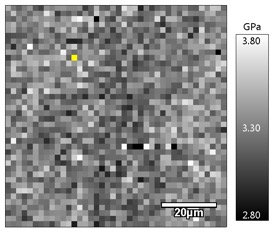 | Elastic modulus histogram 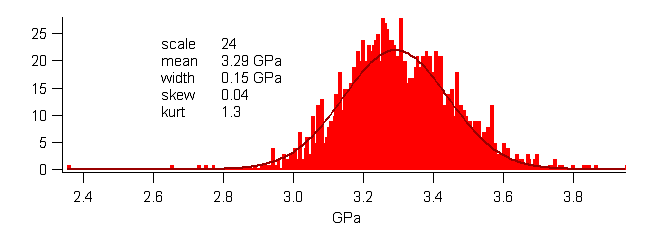 |
| 4. | |
| 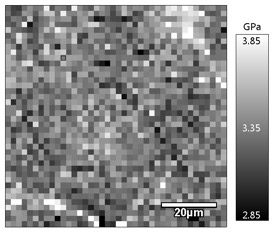 | Elastic modulus histogram 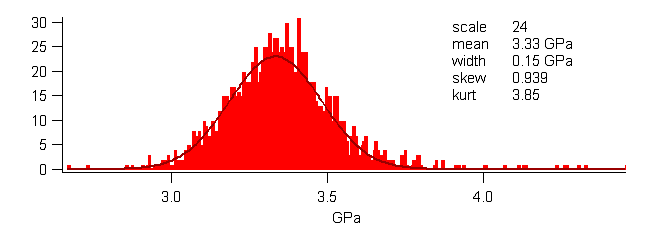  Indentation depth 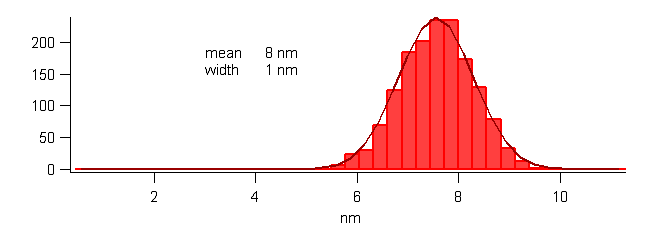 |

Table S7. Blend of 91 kDa and 200 kDa polystyrene; 75/25 w/w%; Rh 0%.(Image processing software: Igor Pro 6.37 with Asylum Research 15.02.105 add-on, https://afm.oxinst.com)

| 1. | |
| --- | --- |
| 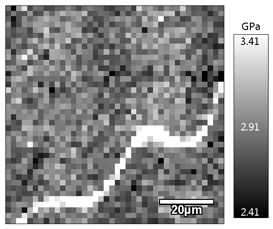 | Elastic modulus histogram 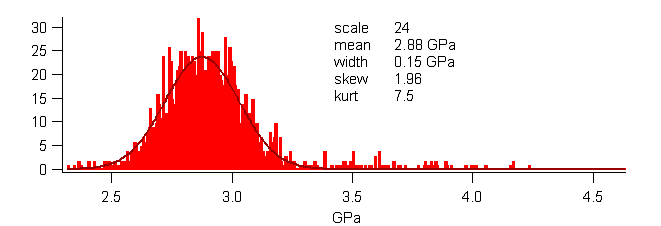  Indentation depth 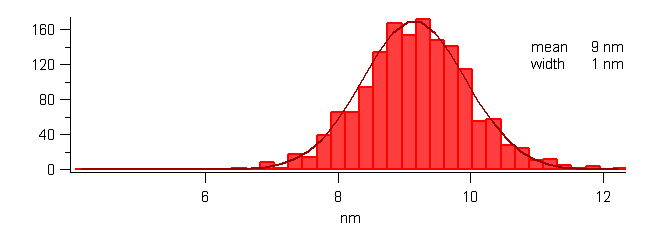 |
| 2. | |
| 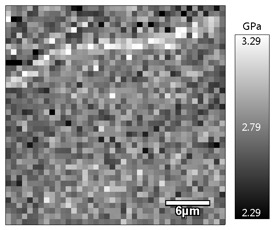 | Elastic modulus histogram 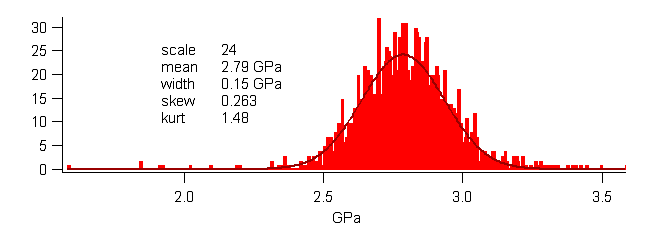 |

Table S8. Blend of 91 kDa and 200 kDa polystyrene; 50/50 w/w%; Rh 0%.(Image processing software: Igor Pro 6.37 with Asylum Research 15.02.105 add-on, https://afm.oxinst.com)

| 1. | |
| --- | --- |
| 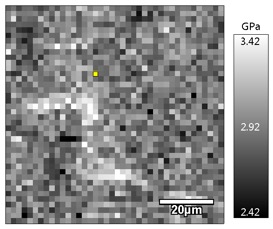 | Elastic modulus histogram 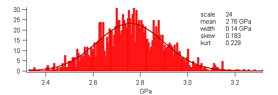  Indentation depth 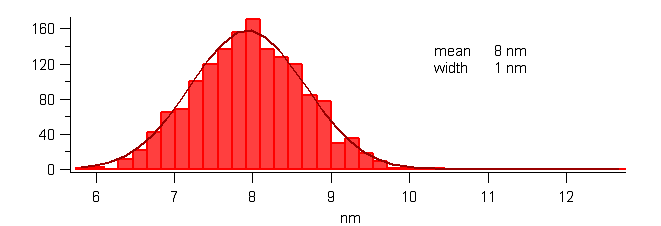 |
| 2. | |
| 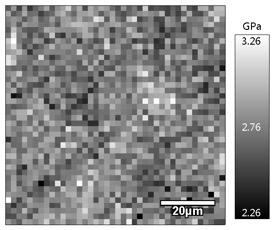 | Elastic modulus histogram  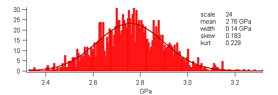  Indentation depth 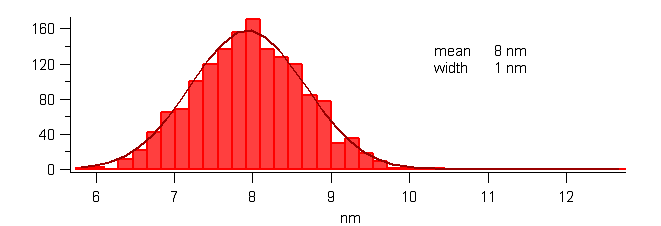 |

Table S9. Blend of 91 kDa and 200 kDa polystyrene; 25/75 w/w%; Rh 0%.(Image processing software: Igor Pro 6.37 with Asylum Research 15.02.105 add-on, https://afm.oxinst.com)

| 1. | |
| --- | --- |
| 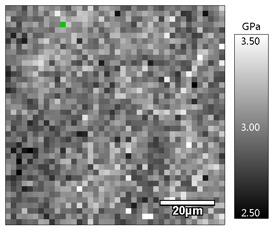 | Elastic modulus histogram  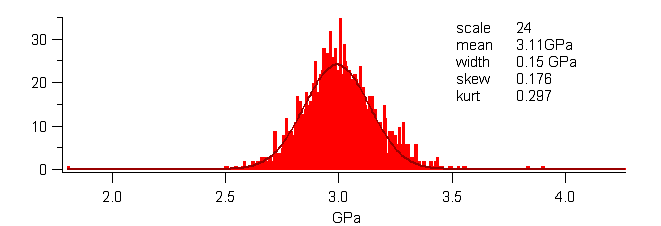 |
| 2. | |
| 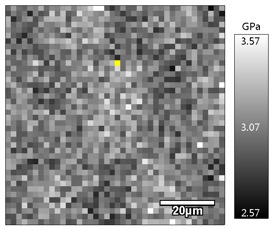 | Elastic modulus histogram  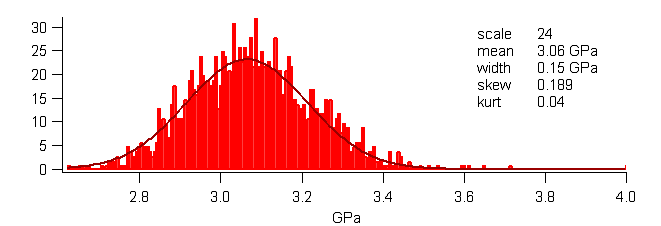 |
| 3. | |
| 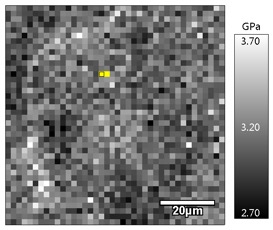 | Elastic modulus histogram  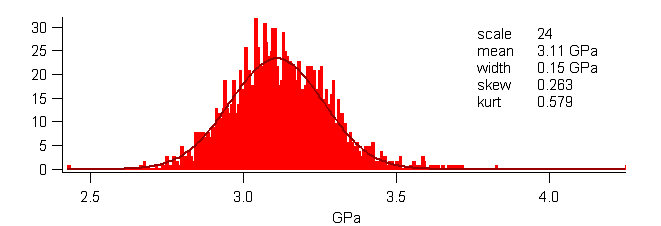  Indentation depth 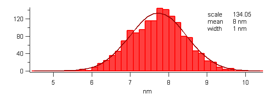 |

Table S10. 20 kDa uniform polystyrene standard; Rh 0%.(Image processing software: Igor Pro 6.37 with Asylum Research 15.02.105 add-on, https://afm.oxinst.com)

| 1. | |
| --- | --- |
| 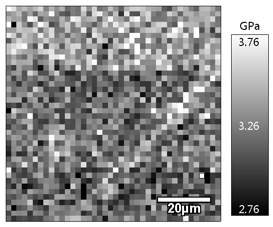 | Elastic modulus histogram  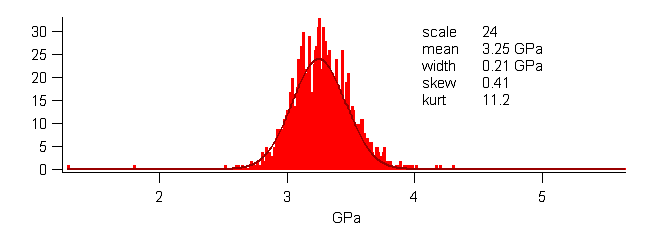  Indentation depth histogram  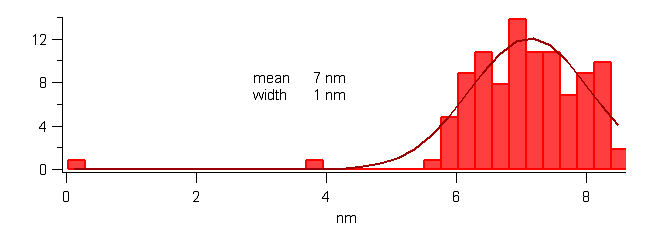 |
| 2. | |
| 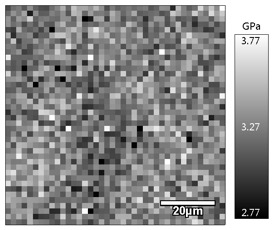 | Elastic modulus histogram  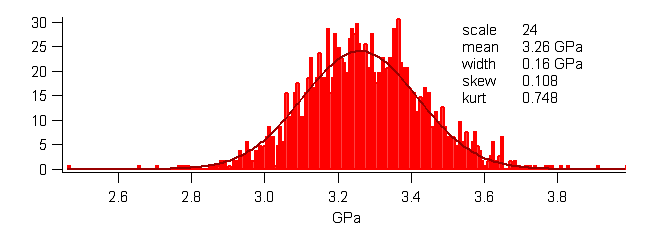 |

Table S11. 91 kDa uniform polystyrene standard; Rh 0%.(Image processing software: Igor Pro 6.37 with Asylum Research 15.02.105 add-on, https://afm.oxinst.com)

| 1. | |
| --- | --- |
| 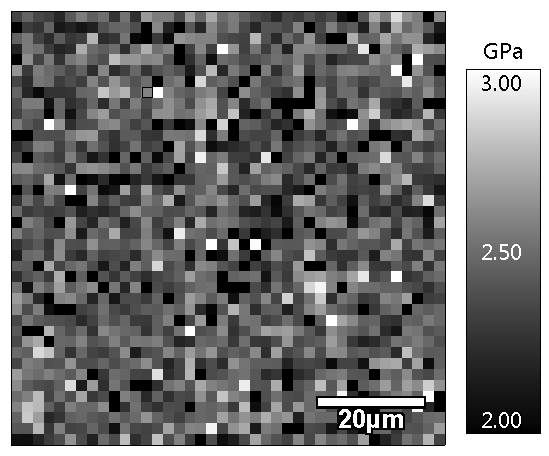 | Elastic modulus histogram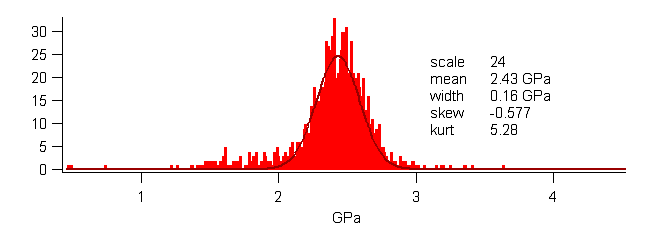  Indentation depth 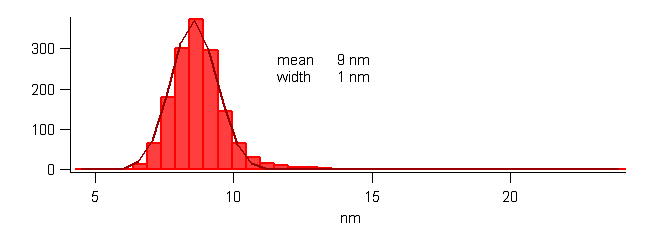 |
| 2. | |
| 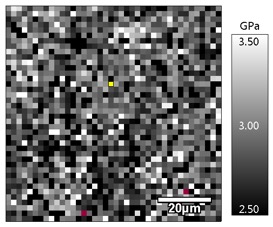 | Elastic modulus histogram  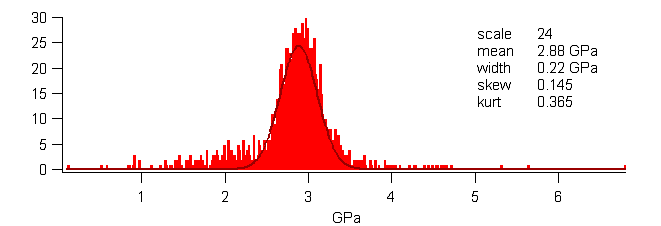 |

Table S12. 150 kDa uniform polystyrene standard; Rh 0%.(Image processing software: Igor Pro 6.37 with Asylum Research 15.02.105 add-on, https://afm.oxinst.com)

| 1. | |
| --- | --- |
| 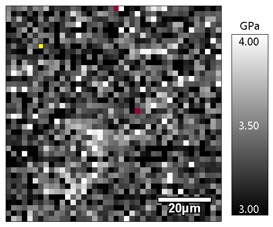 | Elastic modulus histogram  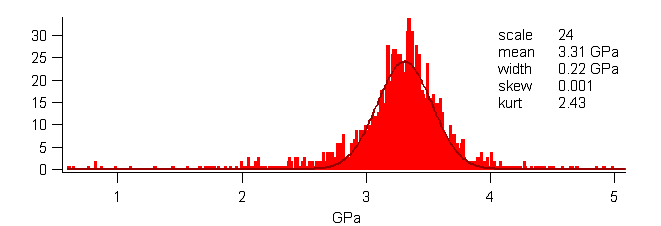  Indentation depth 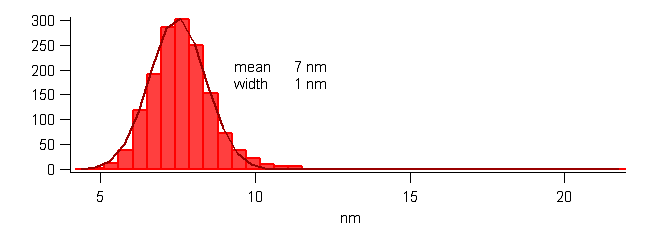 |

Table S13. Blend of 200 kDa uniform polystyrene standard; Rh 0%.(Image processing software: Igor Pro 6.37 with Asylum Research 15.02.105 add-on, https://afm.oxinst.com)

| 1. | |
| --- | --- |
| 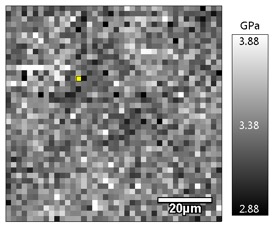 | Elastic modulus histogram  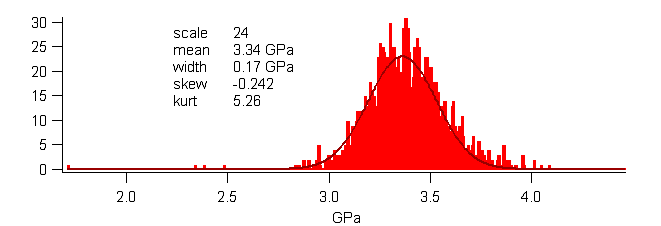  Indentation depth 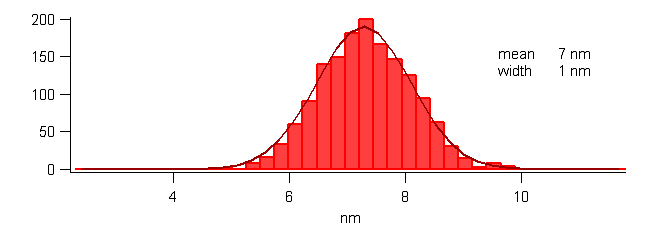 |
| 2. | |
| 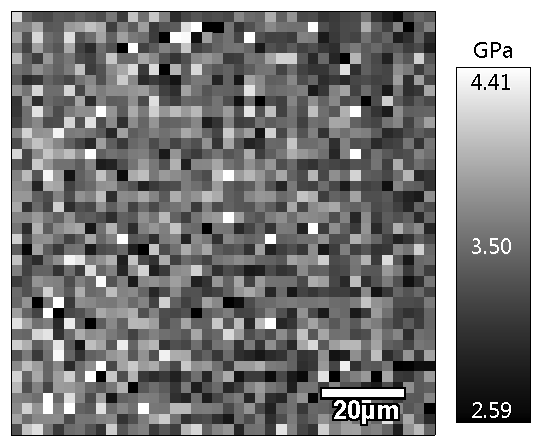 | Elastic modulus histogram  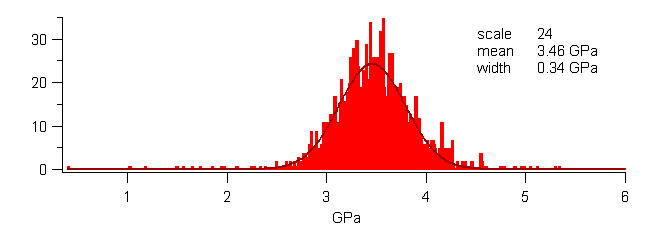 |

Table S14. Mean elastic modulus in GPa. The calculation takes into account all prepared force maps, including higher (images above) and low-resolution maps (images not included).

| Uniform | | | | |  |
| --- | --- | --- | --- | --- | --- |
|  |  |  |  |  |  |
| Type of polystyrene | PS 20 kDa | PS 91 kDa | PS 150 kDa | PS 200 kDa |  |
| Number of tests | 20† | 8 | 6 | 6 |  |
| Mean | 3.271 | 2.896 | 3.165 | 3.272 |  |
| Std. Deviation | 0.272 | 0.382 | 0.339 | 0.318 |  |
| Std. Error of Mean | 0.061 | 0.135 | 0.138 | 0.130 |  |
| Blend of 90 kDa and 200 kDa | | | | |  |
|  |  |  |  |  |  |
| Ratio w/w% | 75/25* | 50/50* | 25/75* |  |  |
| Number of tests | 3 | 3 | 4 |  |  |
| Mean | 2.833 | 2.777 | 3.003 |  |  |
| Std. Deviation | 0.045 | 0.015 | 0.117 |  |  |
| Std. Error of Mean | 0.026 | 0.009 | 0.059 |  |  |
| Blend of 20 kDa and 200 kDa | | | | |  |
|  |  |  |  |  |  |
| Ratio w/w% | 75/25** | 50/50** | 25/75** |  |  |
| Number of tests | 7 | 5 | 6 |  |  |
| Mean | 3,119 | 3,296 | 3,285 |  |  |
| Std. Deviation | 0,376 | 0,049 | 0,427 |  |  |
| Std. Error of Mean | 0,142 | 0,022 | 0,174 |  |  |

*Ordinary one-way ANOVA test for 10 means: the means are significantly different, P = 0.0254.*

*Underlined are means significantly different (p<0.05), Fisher’s multiple comparisons test.*

† *20 kDa polystyrene coatings were used as a control. After couple of tests of different coatings, the 20 kDa coating was retested to control the consistency of the experiment.*

** - Blends of 91 kDa and 200 kDa polystyrene; ** - blend of 20 kDa and 200 kDa polystyrene; x/x – w/w% ratio of blended homogeneous polystyrenes. [M_n_] = f_1_M_w1_ + f_2_M_w2_, where f w/w. % ratio of polymers.*

Table S15. Skewness of the elastic modulus histograms. Only higher resolution maps (map size: 40 lines x40 points, 80 µm x 80 µm) were considered.

| Uniform | | | | |  |
| --- | --- | --- | --- | --- | --- |
|  |  |  |  |  |  |
| Type of polystyrene | PS 20 kDa | PS 91 kDa | PS 150 kDa | PS 200 kDa |  |
| Mean | 0.16 | -0.76 | -0.07 | 0.02 |  |
| Std. Deviation | 0.30 | 0.26 | 0.08 | 0.27 |  |
| Blend of 90 kDa and 200 kDa | | | | |  |
|  |  |  |  |  |  |
| Ratio w/w% | 75/25* | 50/50* | 25/75* |  |  |
| Mean | 0.98 | 0.22 | 0.17 |  |  |
| Std. Deviation | 1.39 | 0.06 | 0.09 |  |  |
| Blend of 20 kDa and 200 kDa | | | | |  |
|  |  |  |  |  |  |
| Ratio w/w% | 75/25** | 50/50** | 25/75** |  |  |
| Mean | 0.34 | 0,50 | 0.16 |  |  |
| Std. Deviation | 0.24 | 0.37 | 0.22 |  |  |

** - Blends of 91 kDa and 200 kDa polystyrene; ** - blend of 20 kDa and 200 kDa polystyrene; x/x – w/w% ratio of blended homogeneous polystyrenes. [M_n_] = f_1_M_w1_ + f_2_M_w2_, where f w/w. % ratio of polymers.*


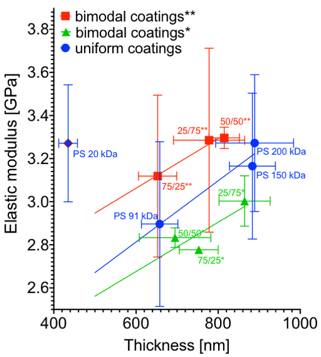


Fig. S4. Regression test between the thickness of the coatings and the elastic modulus. Uniform – coatings were made from homogeneous solutions; Bimodal – coatings made from solutions with bimodal MWD; * - Blends of 91 kDa and 200 kDa polystyrene; ** - blend of 20 kDa and 200 kDa polystyrene; x/x – w/w% ratio of blended homogeneous polystyrenes. [M_n_] = *f_1_M_w1_ + f_2_M_w2_*, where *f* w/w. % ratio of polymers. (Image processing software: GraphPad Prism 9, https://www.graphpad.com/scientific-software/prism/)

Table S16. Are the slops of the linear regression between the thickness of the coatings and the elastic modulus significantly different than zero?

| Is slope significantly non-zero? | | | |
| --- | --- | --- | --- |
| Type of coating | Uniform | ** | * |
| F | 14.25 | 37.15 | 2.314 |
| DFn, DFd | 1, 1 | 1, 1 | 1, 1 |
| P value | 0.165 | 0.104 | 0.370 |
| Deviation from zero? | Not Significant | Not Significant | Not Significant |

** - Blends of 91 kDa and 200 kDa polystyrene; ** - blend of 20 kDa and 200 kDa polystyrene; x/x – w/w% ratio of blended homogeneous polystyrenes. [Mn] = f1Mw1 + f2Mw2, where f w/w. % ratio of polymers.*

1. **Optical imaging.**

|  | 1 | 2 | 3 | 4 |
| --- | --- | --- | --- | --- |


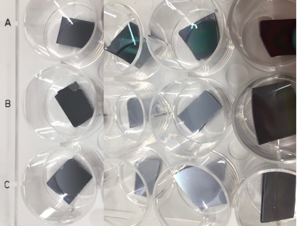


Fig. S5 Example photographies of wafers coated with polystyrene films; First Column: uniform 200 kDa coating; Rows A2 to A4: uniform 20 kDa coating; B2 – C4 – bimodal coatings, 20 kDa and 200 kDa blends; B2, C2: 25/75 w/w; B3, C3: 50/50 w/w; B4, C4: 75/25 w/w.

**
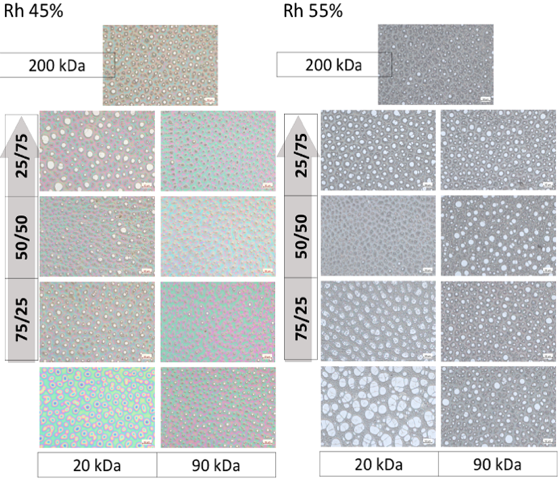
**

Fig. S6**.** Optical microscope images of the coatings prepared from the uniform solutions 20 kDa, 90 kDa and 200 kDa or the bimodal solutions: 75/25, 50/50 or 25/75 w/w %; humidity 45% or 55%. Scalebar is 50 micrometers. (Image processing software: PowerPoint 365, https://office.live.com)


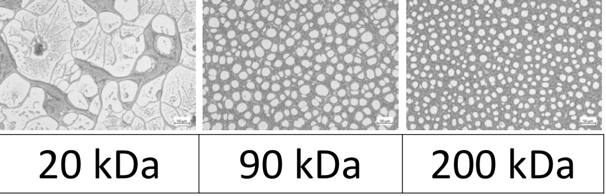


Fig. S7. Optical microscope images of the coatings prepared from the uniform solutions with molecular weight of polystyrene: 20 kDa, 90 kDa and 200 kDa, respectively; humidity Rh 75%. Scalebar is 50 micrometers. (Image processing software: PowerPoint 365, https://office.live.com)


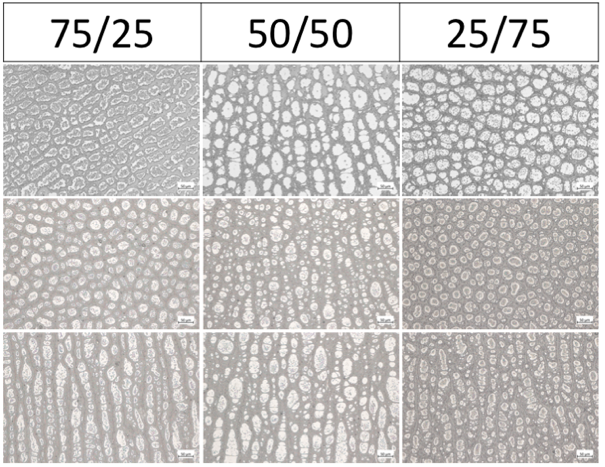


Fig. S8. Optical microscope images of the bimodal coatings prepared from 20 kDa and 200 kDa blends, humidity 75%, molecular weight ratio w/w: 75/25, 50/50 and 25/75, respectively. The first row represents a different set of samples than the next two rows. In the case of second and third two different spots of the same sample are shown in each column. Scalebar is 50 micrometers. (Image processing software: PowerPoint 365, https://office.live.com)


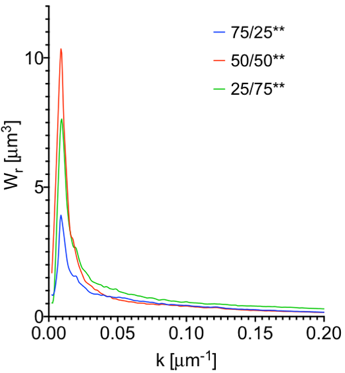


Fig. S9. Radially averaged Powers Spectra Density of coatings images, comparison between coatings made from different ratios of 20 kDa and 200 kDa polystyrene at humidity Rh 75%. Spectra were made by averaging 4 images. (GraphPad Prism 9, https://www.graphpad.com/scientific-software/prism/)

1. **Fourier Transform IR spectroscopy of the blends**

**
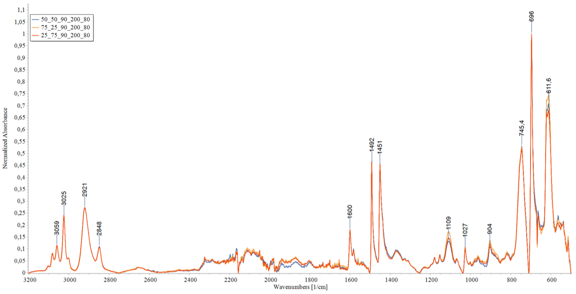
**

Fig. S10. Fourier Transform IR spectra of 90 kDa and 200 kDa coatings on SiO_2_. Compositions 75/25, 50/50 and 25/75 w/w % were investigated. (Spectragryph v1.5.15, https://www.effemm2.de/spectragryph/)


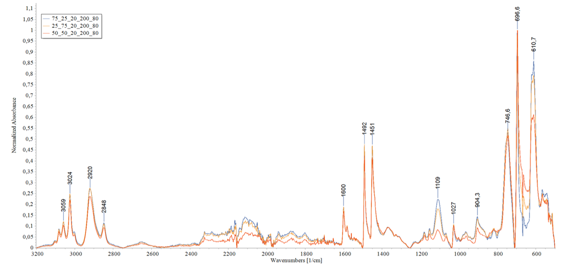
Fig. S11. Fourier Transform IR spectra of 20 kDa and 200 kDa coatings on SiO_2_. Compositions 75/25, 50/50 and 25/75 w/w % were investigated. (Spectragryph v1.5.15, https://www.effemm2.de/spectragryph/)

Fig. S10 and Fig. S11 depicts the FTIR spectra of the 90 kDa and 200 kDa and 20 kDa and 200 kDa coatings, respectively. Coatings spun from 80 mg/ml solutions were investigated. The coatings were spun on SiO_2_ wafers. The vertical axis presents normalized absorbance. The absorbance was normalized to the highest peak. The highest peak was set to 1.

The recognized peaks: 3059 cm^-1^, 3024 cm^-1^ – benzene ring vibrations; 2920 cm^-1^, 2848 cm^-1^ related to -CH_3_ and -CH_2_ groups; 1600 cm^-1^, 1492 cm^-1^ – benzene ring vibrational modes, 1451 cm^-1^: -CH and -CH_2_ groups; 1027 cm^-1^, 904 cm^-1^ - benzene ring vibrational modes; 747 cm^-1^ and 697 cm^-1^ – main benzene vibrational modes; are related to polystyrene chemical structure.[4] Two odd peaks: at 1109 cm^-1^ and 611 cm^-1^ should be attributed to SiO_2_ background as shown in Fig. S12.

**
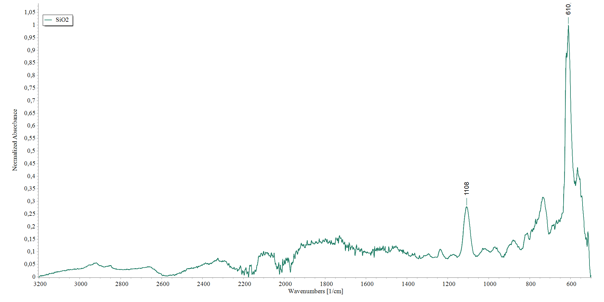
**

Fig. S12. FTIR spectra of SiO_2_ wafer. (Spectragryph v1.5.15, https://www.effemm2.de/spectragryph/)

It can be noticed that the intensity of the 1109 cm^-1^ and 611 cm^-1^ is related to the thickness of the films. The 20 kDa and 200 kDa 75/25 w/w % coating was the thinnest.

To summarize, spectra typical for pristine polystyrene were found. It can be concluded that the end groups were typical -CH_3_ groups.

1. **Surface free energy and contact angle**


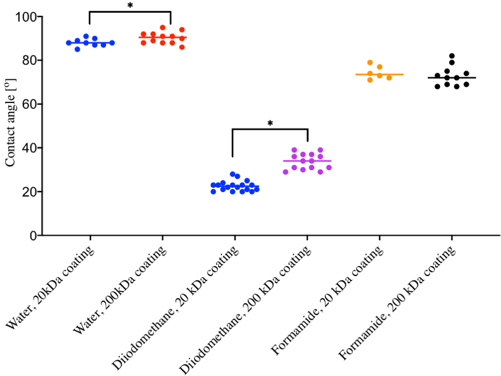


Fig. S13. Contact angle of water, diiodomethane and formamide on 20 kDa and 200 kDa coatings. * - means are significantly different, t test (p < 0.05). (GraphPad Prism 9, https://www.graphpad.com/scientific-software/prism/)


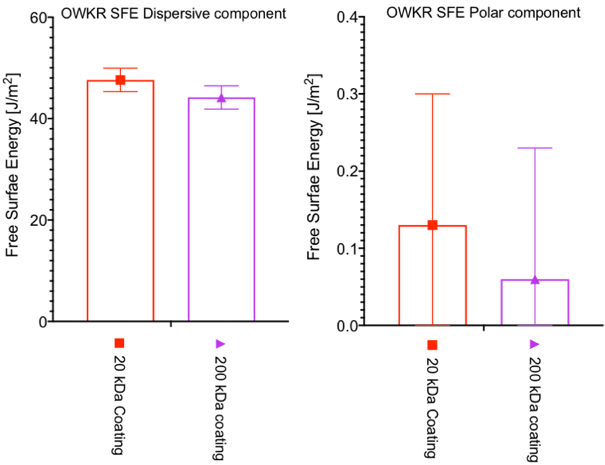


Fig. S14. Free Surface Energy of 20 kDa coating and 200 kDa coating, OWKR Method.[5] (GraphPad Prism 9, https://www.graphpad.com/scientific-software/prism/)

1. **Varying the spin-coating time**


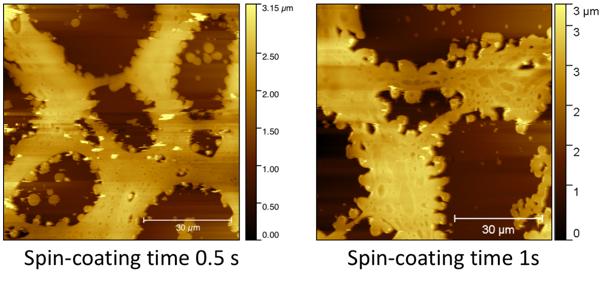
Fig. S15. PS 20 kDa and 200 kDa blend, 75/25 ratio, Spin-coating time: 0.5s and 1s. (Gwyddion v2.50, http://gwyddion.net)

**References:**

[1] Morgan Stefik, “Spincoater,” 2018. [Online]. Available: http://www.stefikgroup.com/spin-coater/.

[2] C. Huang, “Phase Separation in Thin Polymer Films : From Self Stratification to Polymer Blend Lithography,” no. August 2014.

[3] D. T. W. Toolan, “ Straightforward technique for in situ imaging of spin-coated thin films ,” *Opt. Eng.*, vol. 54, no. 2, p. 024109, 2015, doi: 10.1117/1.oe.54.2.024109.

[4] C. Y. Liang, S. Krimm, S. M. Barber-Meyer, L. D. MeCh, C. Y. Liang, and S. Krimm, “Infrared spectra of high polymers. VI. Polystyrene,” *J. Polym. Sci.*, vol. 27, no. 115, pp. 241–254, 1958, doi: doi:10.1002/pol.1958.1202711520.

[5] J. W. Drelich, “Contact angles: From past mistakes to new developments through liquid-solid adhesion measurements,” *Adv. Colloid Interface Sci.*, vol. 267, pp. 1–14, 2019, doi: 10.1016/j.cis.2019.02.002.
